# Enhancing the Oral Bioavailability of Glutathione Using Innovative Analogue Approaches

**DOI:** 10.3390/pharmaceutics17030385

**Published:** 2025-03-18

**Authors:** Naibo Yin, Paul W. R. Harris, Mengyang Liu, Jianguo Sun, Guanyu Chen, Jingyuan Wen, Margaret A. Brimble

**Affiliations:** 1 School of Pharmacy, Faculty of Medical and Health Sciences, The University of Auckland, 85 Park Road, Grafton, Auckland 1023, New Zealand; n.yin@auckland.ac.nz (N.Y.); m.liu@auckland.ac.nz (M.L.); 2 School of Biological Science, Faculty of Sciences, The University of Auckland, Auckland 1010, New Zealand; paul.harris@auckland.ac.nz; 3 Maurice Wilkins Centre for Molecular Biodiscovery, The University of Auckland, Auckland 1142, New Zealand; 4 Key Laboratory of Drug Metabolism and Pharmacokinetics, State Key Laboratory of Natural Medicines, China Pharmaceutical University, Nanjing 210009, China; jgsun@cpu.edu.cn; 5 School of Pharmaceutical Sciences (Shenzhen), Shenzhen Campus of Sun Yat-sen University, Shenzhen 518063, China; chengy239@mail.sysu.edu.cn; 6 School of Chemical Science, Faculty of Sciences, University of Auckland, Auckland 1010, New Zealand

**Keywords:** glutathione (GSH), oral bioavailability, chemical modification, antioxidant, analogues, peptide synthesis

## Abstract

**Background:** Glutathione (GSH) is an essential antioxidant that protects against oxidative stress, but its oral bioavailability is below 1% due to enzymatic degradation and poor gastrointestinal absorption. Improving the oral bioavailability of GSH could significantly enhance its therapeutic efficacy. **Methods:** This study synthesised GSH analogues with chemical modifications to improve bioavailability. Seven GSH derivatives were designed: three analogues with altered stereochemistry (**1.62**, **1.63**, and **1.64**) and three *N*-methylated derivatives (**1.65**, **1.70**, and **1.71**), alongside a native GSH (**1.61**). The analogues were synthesised via Fmoc-solid-phase peptide synthesis, and they were characterised using reverse-phase high-performance liquid chromatography (RP-HPLC), electrospray ionisation mass spectrometry (ESI-MS), Fourier-transform infrared spectroscopy (FTIR), and nuclear magnetic resonance (NMR) spectroscopy. Their toxicity was assessed on Caco-2 cells for viability, and their antioxidant activity was assessed on UVA-irradiated fibroblast cells, enzymatic resistance, and interactions with GSH-metabolising enzymes. **Results**: Among the tested analogues, the *N-*methylated cysteine Compound (1.70) emerged as the most promising candidate. Compound **1.70** demonstrated superior resistance to enzymatic degradation, as well as showing enhanced cell viability and improved antioxidant activity. In vivo studies revealed a 16.8-fold increase in plasma half-life (t½) and a 16.1-fold increase in oral bioavailability compared to native GSH. **Conclusions**: Chemical modification strategies, particularly the *N-*methylation of GSH, present a viable approach to enhancing oral bioavailability. Compound **1.70** showed significant potential for therapeutic applications, warranting further investigation and development in clinical settings.

## 1. Introduction

Peptides are short chains of amino acid monomers, and they are typically no more than 50 residues linked together via peptide bonds [1]. Peptides are popular drug candidates as they generally exhibit less side effects due to their more specific activity when compared to small molecule drugs [2]. Native peptide drugs, however, are generally ineffective when given orally due to low oral bioavailability, which is as a result of the physical and biochemical barriers of the gastrointestinal tract (GIT), often forcing the use of invasive parental delivery methods that lead to low patient compliance [3,4]. Therefore, overcoming the barriers to improve peptide drug oral bioavailability is critical to their successful performance on the market.

Glutathione (GSH) is an important detoxification tripeptide and antioxidant of the human body that consists of the amino acids *γ*-glutamic acid, cysteine, and glycine. The interactions between GSH and the enzymes glutathione peroxidase (GPx) and glutathione reductase (GR) with GSH combats oxidative stress [5,6]. GSH, also known by the IUPAC name (2S)-2-Amino-4-[16]butanoic acid, is highly water-soluble (292.5 mg/mL), with a logP of −6.4 [6]. This high hydrophilicity contributes to the low oral bioavailability of GSH, as it cannot cross the physical barriers of the gastrointestinal tract (GIT). The biochemical barriers of the GIT also include enzymes and microbes that specifically break down the peptide bonds present in all proteins and peptides [7]. A biochemical barrier that is more specific to GSH is the enzyme glutathione S-transferase (GST), which degrades GSH, causing reduced oral bioavailability via first pass metabolism while also reducing T½ [8].

Oxidative stress is caused by reactive oxygen/nitrogen species (ROS/RNS), as well as free radicals that can cause lipid peroxidation, DNA damage, protein oxidation, and other undesirable adverse reactions with various cellular components [9,10]. These negative effects can ultimately lead to an increased risk or progression of the diseased state of many degenerative diseases, such as diabetes, rheumatoid arthritis, cancer, and aging; various neurodegenerative diseases, including Parkinson’s disease and Alzheimer’s disease; and various cardiovascular diseases, including heart failure, myocardial infarction, and arteriosclerosis [11,12,13,14,15]. Additionally, the interaction of GSH with the key enzyme glutathione S-transferase (GST) conjugates GSH to toxins in our body for elimination [16,17]. GSH, however, is rapidly hydrolysed by the hepatic enzyme γ-glutamyl transferase (GGT), which, in addition to the biological barriers of the GIT, accounts for its low oral bioavailability [18].

Drug analogues are compounds that are similar to the parent drug but are chemically modified to enhance certain characteristics while retaining its biological activity [19,20]. Of the many possible modifications tested in our preliminary studies, two stand out chemical modifications used for enhancing peptide bioavailability were found to change the stereochemistry of its amino acid constituents and *N-*methylation [21,22]. Altering the α-carbon stereochemistry (from L to D) can make peptide analogues resistant to some enzymes while still being recognisable by others. A functional peptide analogue with improved oral bioavailability can be fabricated by retaining specificity for the enzymes responsible for its bioactivity while decreasing specificity to the enzymes associated with its degradation [23]. *N-*methylation, on the other hand, adds a small methyl group to the nitrogen of the peptide bond. The use of a methyl group is optimum in that it is small enough to not influence the overall activity of the molecule, while the rationale behind the location of the modification is to protect the most common degradation location of the peptides, i.e., the peptide bond. The addition of a methyl group to nitrogen also reduces the number of hydrogen bond acceptors by 1, reducing hydrophilicity [9,24,25]. The aim of this research was to synthesise an orally available GSH analogue with retained efficacy by improving its stability. This study builds on and applies theory in peptide drug development to increase access to the variety of health benefits that GSH is known to provide.

## 2. Materials and Methods

### 2.1. Materials

All of the reagents were used as supplied. The 2-(1H-Benzotriazol-1-yl)-1,1,3,3-tetramethyluronium hexafluorophosphate (HBTU); 4-(hydroxymethyl)phenoxyacetic acid (HMP); *N*-(9-fluorenylmethoxycarbonyl)-L-glycine (Fmoc-L-Gly-OH); *N*-(9-fluorenylmethoxycarbonyl)-L-cysteine (trityl) (Fmoc-L-Cys(Trt)-OH); *N*-(9-fluorenylmethoxycarbonyl)-L-glutamic acid (tert-butyl) (Fmoc-L-Glu-OtBu); *N-*(9-fluorenylmethoxycarbonyl)-D-cysteine (trityl) (Fmoc-D-Cys(Trt)-OH); *N*-(9-fluorenylmethoxycarbonyl)-D-glutamic acid (tert-butyl) (Fmoc-D-Glu-OtBu); and *N*-(9-fluorenylmethoxycarbonyl)sarcosine (Fmoc-Sar-OH) (≥98% purity) were purchased from GL Biochem (Shanghai, China). The *N,N*’-Diisopropylethylamine (DIPEA); triisopropylsilane (TIPS); piperidine; methanol; ethanol; 1,2-ethanedithiol (EDT); 2-mercaptoethanol; 2-nitrobenzenesulfonyl chloride (o-NBS-Cl); and 1,8-diazabicyclo[5.4.0]undec-7-ene (DBU) were purchased from Sigma-Aldrich (St. Louis, MO). The dichloromethane (DCM), dimethylformamide (DMF), and acetonitrile (ACN) were purchased from Scharlau (Barcelona, Spain), and the trifluoroacetic acid (TFA) was purchased from Oakwood Chemicals (West Columbia, SC). Unless otherwise stated, all of the reactions occurred under atmospheric conditions. The sodium hydroxide (NaOH); hydrochloric acid (HCl); reduced glutathione (GSH); pepsin from bovine; trypsin from bovine; chymotrypsin from bovine; aminopeptidase γ,γ-glutamyl transferase (GGT) from equine kidney type VI; glutathione reductase (GR) from yeast; glutathione peroxidase (GPx) from bovine erythrocytes; glutathione S-transferase (GST) from equine liver; 1-chloro-2,4-dinitrobenzene (CDNB); nicotinamide adenine dinucleotide phosphate (NADPH); Tris(hydroxymethyl)aminomethane (Tris) buffer; ethylenediaminetetraacetic acid (EDTA); and tert-butyl hydroperoxide (tBu-OOH) were purchased from Sigma-Aldrich. Hank’s balanced salt solution (HBSS) and phosphate-buffered saline (PBS) were purchased from Thermo Fisher Scientific (Auckland, New Zealand). The rat jejunum luminal contents and mucosal homogenates were extracted from euthanised rats at the University of Auckland (12–18 months prior). The caco-2 cell line was purchased from the American Type Culture Collection (Manassas, VA, USA). Dulbecco’s modified Eagle’s medium (DMEM) with high glucose, non-essential amino acid (NEAA) solution; foetal bovine serum (FBS); penicillin-streptomycin; and trypsin-EDTA were purchased from Invitrogen (Auckland, New Zealand). The 3-[4,5-dimethylthiazol-2-yl]-2,5-diphenyl tetrazolium bromide (MTT) was sourced from Sigma-Aldrich. Milli-Q water was used throughout the experiments. The scaled-up synthesis of Compound 1.70 (purity > 95%) was ordered from DGpeptides Co., Ltd. (Wuhan, China). All the other reagents used were of analytical grade.

### 2.2. Synthesis of Glutathione and Analogues

**Figure 1:**
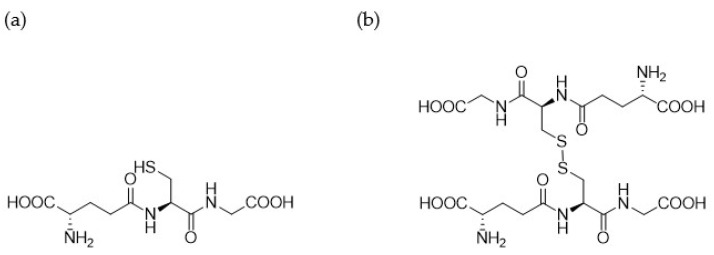
The chemical structure of (**a**) reduced GSH and (**b**) oxidised GSH (GSSG).

Compounds **1.62** (D-cysteine modified), **1.63** (D-glutamic acid modified), **1.64** (both D-cysteine and D-glutamic acid modified), **1.65** (*N*-methylated glycine), **1.70** (*N*-methylated cysteine), and **1.71** (*N*-methylated glutamic acid) were synthesised, characterised, and screened in comparison to Compound **1.61** (glutathione synthesised under the same conditions) and commercially available glutathione. The reduced and oxidised forms of glutathione that were used are shown in Figure 1, and a list of the structures for all synthesised analogues are shown in Table 1.

The native Compound **1.61**, as well as Compounds **1.62**, **1.63**, **1.64**, and **1.65** (as shown in Table 1), were synthesised by manual solid phase peptide synthesis (SPPS) in a glass reaction vessel on aminomethylated polystyrene resin at a 0.5 mmolg^−1^ scale [26,27]. SPPS was chosen as the synthetic method as it can produce a large variety of modified peptides for screening by changing the building blocks (Fmoc-AA-OH) used. The HMP linker (2 eq.) and DIC (1.1 eq.) were dissolved in a minimum amount of DCM:DMF (4:1 *v*/*v*); they were then attached to pre-swollen aminomethylated polystyrene resin (0.549 g) of loading capacity 0.91 mmolg^−1^ and agitated for 2 h. The resin was then drained and washed with DCM and DMF. Compounds **1.70** and **1.71** were also synthesised in a similar manner but with additional *N*-methylation steps performed on their respective amino acids.

The first amino acid (3 eq.) and DIC (1.1 eq.) was dissolved in a minimum amount of DCM:DMF (4:1 *v*/*v*) and added to the linker on resin followed by a drop wise addition of a catalytic amount of 4-dimethylaminopyridine (DMAP) (0.16 mmol), which was dissolved in DCM and then agitated for 2 h. The resin was drained and washed with DCM and DMF. The above steps were repeated three times to ensure reaction completion.

The base-labile Fmoc group was removed from the N-terminus of the growing peptide chain by agitation with 20% *v*/*v* piperidine in DMF (15 mL) for 5 min, which was then drained and repeated for a further 15 min. The resin was then drained and washed with DMF. Subsequent amino acids were coupled to the N-terminal free amine. Fmoc-AA-OH (5 eq.), HBTU (4.8 eq.), and DIPEA (10 eq.) were dissolved in DMF and agitated with the resin for 1 h. The resin was then drained and washed with DMF. The Fmoc group was then removed according to the method above, and the final amino acid was attached using the same method as the second amino acid.

**Figure 2:**
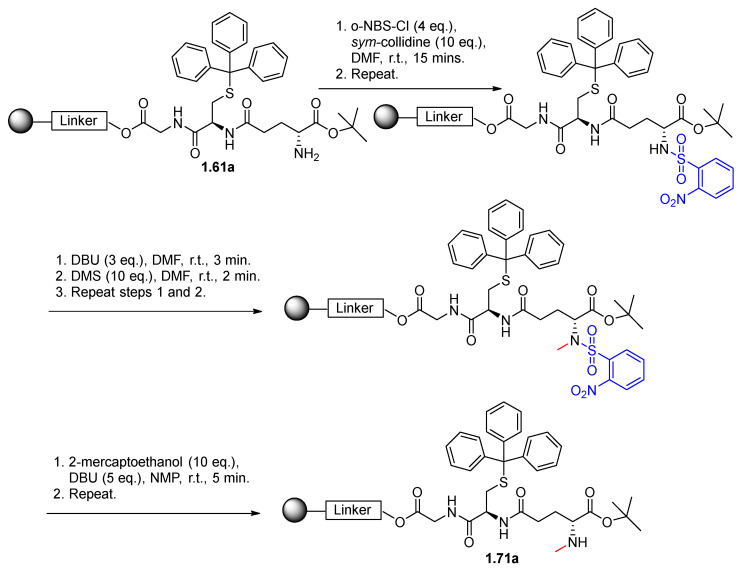
Scheme of the N-methylation of amino acids (created by ChemBioDraw Version 15.0) (modification highlighted in red, protecting group highlighted in blue).

*N*-Methylation of Compounds **1.70** and **1.71** was achieved using the steps outlined in Figure 2 on resin using 1,8-diazabicyclo(5.4.0)undec-7-ene (DBU). The free amine group of cysteine or glutamic acid was first protected using 2-nitrobenzenesulfonyl chloride (o-NBS-Cl), which prevents double *N*-methylation and can be later removed with 2-mercaptoethanol following *N*-methylation using dimethylsulfate (DMS) [28].

The peptide sequence attached to the HMP linker was both side-chain deprotected and cleaved from resin by agitation with a mixture of 92.5:2.5:2.5:2.5 TFA:TIPS:EDT:H_2_O (*v*/*v*/*v*/*v*, 5 mL) for 2 h. The resin was filtered, the filtrate was collected, and it was then evaporated under nitrogen. Following cleavage, the peptide was precipitated by the addition of diethyl ether (50 mL). The peptide was collected by centrifuge and the diethyl ether was decanted and discarded. The wash was repeated three times. The peptide was reconstituted in a mixture of water and ACN with 0.1% TFA. A small amount of sample was withdrawn for HPLC and MS analysis, the remaining solution was freeze-dried, and then the solid powder stored under N_2_ at −20 °C until use.

### 2.3. Characterisation and Purification

#### 2.3.1. Reverse-Phase High-Performance Liquid Chromatography

Analytical reverse-phase high-performance chromatography (RP-HPLC) was performed on a Dionex Ultimate 3000 system using the following column: Grace Vydac Denali C18, 120 Å, 100 × 2.1 mm, 5 μm. A linear gradient of 0.1% TFA in water (Solvent A) and 0.1% TFA in acetonitrile (ACN) (Solvent B) was used, starting at 99% A with 1% B and increasing per minute at a flow rate of 1 mL/min and detection at 215 nm. Samples were dissolved in a 50:50 mixture of water and ACN with 0.1% TFA. RP-HPLC purification was conducted by collection under the same conditions on a semi-prep scale using a Grace Vydac Denali C18, 120 Å, 150 × 10.00 mm, and 5 μm column at a 5 mL/min flow rate.

#### 2.3.2. Electrospray Ionisation Mass Spectra

Electrospray ionisation mass spectra (ESI-MS) was recorded on an Agilent 6120 Quadruple mass spectrometer with an Agilent 1260 Infinity liquid chromatography system (Santa Clara, CA, USA). The samples dissolved in a mixture of water and ACN with 0.1% TFA were introduced using direct flow injection at 0.2 mL/min into an ESI source in positive or negative mode.

#### 2.3.3. Fourier Transformed Infra-Red Spectroscopy

Fourier transformed intra-red spectroscopy (FTIR) was performed on a Tensor 37 FT-IR spectrometer (Bruker Optics, Ettlingen, Germany) system equipped with an attenuated total reflection Ge crystal cell in the reflection mode. Background signals were recorded and subtracted automatically from the sample reading. The sample was scanned with 64 accumulations to reduce background noise. The wavenumber range was recorded from 400 to 4000 cm^−1^ with a resolution of 4 cm^−1^. Samples were measured in powder form.

#### 2.3.4. Nuclear Magnetic Resonance

Nuclear magnetic resonance was performed on an AVIII-400 ICON automation spectrometer. Then, ^1^H, ^13^C, Dept 135, and Dept 90 experiments were conducted on the final products with 16, 400, 200, and 200 accumulations, respectively. The samples were dissolved in pure heavy water or a mixture of heavy water and ACN. All chemical shifts (δ) are reported in parts per million (ppm) relative to the tetramethylsilane peak, which was defined as δ 0.00 ppm, or as relative to residual chloroform δ 7.26 for 1H NMR and δ 77.1 ppm for 13C NMR.

### 2.4. Biological Screening

#### 2.4.1. Stability

To investigate the potential peptide degradation during storage and when in a cell culture medium, the stability of GSH in NaOH (0.1 molL^−1^, pH 13.0), HBSS (pH 7.4), and HCl (0.1 molL^−1^, pH 1.0) were investigated. GSH (100 μgmL^−1^) was incubated at 37 °C with the above conditions for 24 h or until at least 20% of peptide was degraded. The samples were withdrawn at the time intervals 0, 1, 10, 30, 60, 120, 240, 480, and 1440 min and mixed with equal volume of ice-cold HCl (0.2 molL^−1^) until analysis by HPLC. Stability was repeated until three results within <5% SD were obtained.

#### 2.4.2. Enzymatic and Cellular Degradation

To investigate the GSH degradation against common enzymes of the GI tract, the degradation of GSH in the presence of pepsin, chymotrypsin, trypsin, aminopeptidase, GGT, jejunum luminal content, and mucosal homogenates obtained from the rats’ intestines were investigated. GSH (100 μgmL^−1^) was incubated at 37 °C with the above enzymes or mixtures for 24 h. The sample was withdrawn at the time intervals of 0, 1, 10, 30, 60, 120, 240, 480, and 1440 min, which were then mixed with equal volume of ice-cold HCl (0.2 molL^−1^) until analysis by HPLC. Enzymatic degradation was repeated until three results within <5% SD were obtained.

Degradations of GSH were assessed in both a Caco-2 cell culture system, as well as a Caco-2/HT-29 co-cultured system. Caco-2 cells (100,000 cells/mL, 5 mL) were seeded into 100 mm diameter round cell culture dishes and grown under standard cell culture conditions until 90% confluence.

Next, 100 μL of each novel compound and GSH (0.0315 molL^−1^) were added to each dish containing pH = 7.4 PBS (800 μL, pH 9.0). The cells were incubated at 37 °C while shaking for the entire duration of the experiment. The reaction was initiated by the addition of a GGT enzyme solution (100 μL, 10 UmL^−1^). Aliquots of 50 μL were removed from the reaction vessel at 0, 1, 10, 30, 60, 120, 240, 480, and 1440 min, and these were then added to cold HCl (50 μL, 0.1 molL^−1^) and kept frozen until analysis using HPLC. HPLC was used to monitor the rate of decrease in the peak area of GSH, which was found to be correlated to the concentration of the peptide remaining. Cellular degradation was repeated until three results within <5% SD were obtained.

#### 2.4.3. γ-Glutamyl Transferase Degradation

Each compound and GSH (100 μgmL^−1^, 0.0315 molL^−1^) was added to each reaction vessel containing phosphate buffer solution (800 μL, pH 9.0). The reaction vessels were incubated at 37 °C with shaking for the entire duration of the experiment. Reaction was initiated by the addition of GGT enzyme solution (100 μL, 10 UmL^−1^). Aliquots of 50 μL were removed from the reaction vessel at the time intervals of 0, 1, 10, 30, 60, 120, 240, 480, and 1440 min, and they were then mixed with an equal volume of ice-cold HCl (0.2 molL^−1^) until analysis using HPLC. The degradation rate of the novel compounds analysed by HPLC were compared with that of GSH. GGT degradation was repeated until three results within <5% SD were obtained. A GGT degradation less than 50% was considered resistance to GTT activity [29].

#### 2.4.4. Glutathione S-Transferase Assay

The degree of conjugation of GSH or novel compounds to a substrate catalysed by GST can be monitored flourometrically by conjugation with the substrate 1-chloro-2,4-dinotrobenzene (CDNB) to a thiol group. Successful conjugation results in the formation of a S-DNB conjugate, which strongly absorbs at 340 nm [30,31,32].

A stock solution of GSH and novel compounds (0.0315 mmolL^−1^) were prepared. GSH or novel compounds (26 µL) were added to a mixture of pH = 7.4 DPBS (170 µL), CDNB (2 µL, 100 mmolL^−1^), and GST enzyme (2 µL, 25 UmL^−1^) to initiate the reaction. The rate of increase in absorbance at 340 nm was monitored every minute over a 10 min period. The rate of increase in absorbance at 340 nm was calculated by the following equation (Equation (1)), where ΔA_340_ is the change in absorption at 340 nm per minute [32]:

(1) $$\Delta A340=\frac{A340(final)–A340(initial)}{t}.$$

The rate of increase in absorption at 340 nm was correlated to the specific activity of GST using the following equation (Equation (2)):

(2) $$\frac{\Delta A340\times V}{\varepsilon \mathrm{mM}\;\times V\mathrm{enz}}=Specific\;activity\;of\;GST,$$

where the extinction coefficient εmM for a 96-well plate of a path length of 0.5 cm is 5.3 mmolL^−1^cm^−1^; Venz is the volume of the GST enzyme sample used (2 µL); and V is the total volume used (200 mL). Enzymatic activity was repeated until three results within <5% SD were obtained. A GST activity more than 50% was considered as retaining GST activity [29].

#### 2.4.5. Glutathione Reductase and Glutathione Peroxidase Assay

The activity of GR can be measured by monitoring the rate of decrease in absorbance of its substrate, NADPH, which absorbs light of 340 nm in the presence of a second substrate, i.e., oxidised GSH. Oxidised GSH can be formed by the oxidation of reduced GSH. In the presence of peroxides, reduced GSH can be oxidised by the enzyme GPx while reducing the peroxide. Therefore, reduced GSH and analogues that have maintained both specificity with both GR and GPx will show a faster decrease in absorption at 340 nm in the presence of GR, GPx, NADPH, and peroxide, as shown in Equations (3) and (4).

(3) $$R\text{-}\mathrm{OOH}+2\mathrm{GSH}\;\overset{\mathrm{GPx}}{\to }R\text{-}\mathrm{OH}+\mathrm{GSSG}+H2O,$$

(4) $$\mathrm{GSSG}+\mathrm{NADPH}+H^{+}\overset{\;\mathrm{GR}\;}{\to }2\mathrm{GSH}+\mathrm{NAD}P^{+}$$

Each compound and GSH (10 μL, 0.0315 molL^−1^) was mixed with Tris•HCl buffer (50 mmolL^−1^, pH 8.0) containing EDTA (148 μL, 0.5 mmolL^−1^). The NADPH solution (10 μL, 5 mmolL^−1^), GPx enzyme solution (10 μL, 0.25 UmL^−1^), and GR enzyme solution (10 μL, 10 UmL^−1^) were also added to the reaction mixture. The reaction was initiated by the addition of tBu-OOH (2 μL, 30 mmolL^−1^), and the decrease in absorbance at 340 nm was recorded by a microplate reader every 10 secs for one minute. The rate of decrease in absorbance at 340 nm was compared to the rate of decrease in absorbance at 340 nm of the blank, which contained no active compound in the Tris•HCl buffer (50 mM, pH 8.0) containing EDTA (158 μL, 0.5 mmolL^−1^), NADPH solution (10 μL, 5 mmolL^−1^), GPx enzyme solution (10 μL, 0.25 UmL^−1^), GR enzyme solution (10 μL,10 UmL^−1^), and tBu-OOH (2 μL, 30 mmolL^−1^). Enzymatic activity was repeated until three results within <5% SD were obtained. A GR and GPx activity more than 50% was considered as retaining GR and GPx activity [29].

#### 2.4.6. Cell Culture

All cell culture studies were conducted in aseptic conditions. Incubation was maintained at an atmosphere of 37.0 °C, 5% CO_2_, and 95% relative humidity. The caco-2 cells were grown in complete Dulbecco’s modified Eagle’s medium (DMEM), which was prepared by adding 10% foetal bovine serum (FBS), 1% penicillin-streptomycin (P-S), and 1% non-essential amino acids (NEAA) to sterile DMEM. Cells were cultured in T-25 and T-75 tissue culture flasks, and the medium was exchanged every 3 days until 90% confluence was reached. Cells were detached and subcultured with 0.25% Trypsin-EDTA. The suspended cell concentration was determined by cell counting.

#### 2.4.7. Cytotoxicity

Cytotoxicity was measured by measuring cell viability using MTT. Then, 90% confluent cells were detached by 0.25% trypsin-EDTA, which were then counted and seeded into sterile 96-well plates. Caco-2 cells were seeded into 96-well plates at a density of 100,000 cells per well and incubated for 24 h prior to the experiment. Fibroblast cells were seeded into 96-well plates at a density of 10,000 cells per well and incubated for 24 h prior to the experiment. HT-29 cells were seeded into 96-well plates at a density of 10,000 cells per well and incubated for 24 h prior to the experiment. The supernatant in each well were discarded and the attached cells were washed using serum-free Fluorobite^TM^ DMEM twice to remove any phenol red. The peptide, at concentrations of 10 mgmL^−1^, 1 mgmL^−1^, 0.1 mgmL^−1^, 0.01 mgmL^−1^, 0.001 mgmL^−1^, and 0.0001 mgmL^−1^ were dissolved in serum-free Fluorobite^TM^ DMEM. The total volume of sample in each well used was 100 μL. The cells were incubated with the peptide for 24 h. Cells were also incubated with serum-free DMEM (positive control) and 2% Triton-X (negative control) for 24 h. The supernatant in each well was then discarded and washed using serum-free Fluorobite^TM^ DMEM. MTT (100 μL, 1.2 mmolL^−1^) dissolved in Fluorobite^TM^ DMEM was then added to each well and incubated for 2 h for MTT conversion into formazan. The supernatant was then discarded, and the cells were washed using Fluorobite^TM^ DMEM to remove any residual MTT and formazan that were not attached to cellular mitochondria. Formazan was dissolved in dimethyl sulfoxide (DMSO) (50 μL), and the absorbance was measured on a microplate reader at a wavelength of 570 nm [6,30,33,34,35,36]. Cell viability was expressed as a percentage of the positive control and calculated as follows (where A_exp_ is the absorbance at 570 nm of the experimental group, A_neg_ is the absorbance at 570 nm of the negative control, and A_con_ is the cell viability of the positive control): Cell viability = (A_exp_ − A_neg_)/(A_con_ − A_neg_).

#### 2.4.8. UV Rescue

Fibroblast cells were seeded into 96-well plates at a density of 10,000 cells per well determined by cell counting. Cells were allowed to settle and attach for 24 h before the addition of peptides. The supernatant in each well was discarded, and the attached cells were washed using serum-free DMEM twice. One group of cells was exposed to UVA under a UV lamp for 1 h. A second group of cells was not exposed to UV irradiation. The supernatant was removed from each well of both groups and replaced with peptide (100 μL, 0.0325 mmolL^−1^), which was then dissolved in Fluorobite^TM^ DMEM. Each plate, including 2 wells of both groups was also incubated with serum-free DMEM (positive control) and 2% Triton-X (negative control) for 24 h. The supernatant in each well of both groups was then discarded and the cells washed using PBS. MTT (100 μL, 6 mmolL^−1^) that was dissolved in HBSS was then added to each well and incubated for 2 h for MTT conversion into formazan. The supernatant was then discarded, and the cells were washed using PBS to remove any residual MTT and formazan that were not attached to the cellular mitochondria. Formazan was dissolved in DMSO (50 μL), and the absorbance was measured on a microplate reader at a wavelength of 570 nm.

#### 2.4.9. In Vivo Study

Pharmacokinetic studies were conducted using a male Sprague Dawley rat model. Male Sprague Dawley rats weighing 200 ± 20 g were purchased from Sippr/*Bk Lab* Animal Co., Ltd. (Shanghai, China) and housed at 25 ± 1 °C with a scheduled 12 h light/dark cycle, and a humidity of 50 ± 10% was set with free access to food and water. The animal experiments were performed in China, complying with the guide for care and use of laboratory animals. The ethics approval was granted by China Pharmaceutical University with the assistance of Dr Jianguo Sun and Dr Guanyu Chen (Ethic number: CPU-2018-DMPK-03-01) (Nanjing, China). In brief, 16 male Sprague Dawley rats were divided evenly between 4 groups (*n* = 4). The first group was administered a GSH solution (control) by intravenous (IV) injection, while the second group was administered a GSH solution by oral gavage. The third group was administered a solution of **1.70** by IV injection, and the fourth group received a solution of analogue **1.70** by oral gavage. Both the oral and IV drug dose given was 75 mg per rat. As there were no cytotoxicity detected in prior studies for Analogue 1.70, the same dose as GSH were given to the rats. The dose used in rats was determined from the acceptable human GSH dose multiplied by the human–rat drug conversion factor of 0.162 [37].

Blood samples were collected into anticoagulant pre-treated tubes at intervals of 0, 5, 15, 30, 60, 120, 240, and 360 min for the IV-treated groups and at the intervals of 0, 30, 60, 120, 240, 360, 1440, and 2880 min for the orally administered groups. The collected blood samples were centrifuged at 7104× *g* for 5 min, and the plasma was stored at −70 °C until analysis. The plasma protein was precipitated by the addition of 1 mL of ACN, which was then vortexed for 1 min and centrifuged at 30,065× *g* for 15 min. The supernatant was filtered, and the GSH concentration in each group (oral/IV) was determined independently using the established LC-MS method, with *m*/*z* transitions of 308.0→179.0 [6]. The **1.70** concentration for each group (oral/IV) was determined using a modified LC-MS method adapted from the GSH protocol. Briefly, blood samples containing **1.70** were analysed separately by positive mode LC-MS using an Agilent 1100 series HPLC (Agilent Technology, Inc., Santa Clara, CA, USA) coupled with an Agilent 6530 Accurate Mass Q-TOF LC/MS (Agilent Technology, Inc., Santa Clara, CA, USA), which was equipped with a dual jet stream electrospray ionisation source. Chromatographic separation was achieved on a Zorbax SB C18 column (5 µm, 3 × 150 mm) maintained at 40 °C. A gradient elution method with initial mobile phase composition of 90:10 water (0.1% formic acid) and acetonitrile (0.1% formic acid) was increased to 30:70 over 1 min and maintained for 2 min. The flow rate was set at 0.5 mL/min with an injection volume of 8 µL. Mass spectrometer settings were configured for *m*/*z* transitions of 319.1→215.2, with a fragmentor voltage of 180 V. The instrument parameters were as follows: a gas flow of 10 L/min, a gas temperature of 275 °C, a sheath gas flow of 12 L/min, a sheath gas temperature of 300 °C, a nebuliser pressure of 40 psi, a capillary voltage of 3000 V, and a nozzle voltage of 500 V. This method was developed and validated through triplicate linearity studies at concentrations ranging from 30 to 5000 ng/mL according to the International Conference on Harmonization (ICH) guidelines [38]. Data were acquired and analysed using Agilent Mass Hunter Software (version A.02.17).

The total area under the serum concentration–time curves from 0 to 48 h (AUC0-48) (nghrmL^−1^) for each group were determined using GraphPad Prism Software version 7.0. The oral bioavailability (F) of GSH and Analogue **1.70** were calculated using the following equation (Equation (5)):

(5) $$F\;(\mathrm{oral})=\frac{AUC\;(oral\;)}{AUC\;(i.v.)}\times 100\%.$$

### 2.5. Statistical Analysis

Data comparisons were conducted using regression analysis, ANOVA tests, and two-tailed *t*-tests. A *p*-value of ≤0.05 was pre-defined as the minimum level of significance. All data were expressed as the mean ± SD, *n* ≥ 3.

## 3. Results and Discussion

### 3.1. Synthesis of ***1.61***–***1.65*** and ***1.70***–***1.71***

Compounds **1.61**–**1.64** and **1.70** were synthesised by manual SPPS in a glass reaction vessel using three different building blocks each on aminomethylated polystyrene resin at a 0.5 mmolg^−1^ scale (see Table 2). The HMP linker (0.182 g, 2 eq.) was attached to aminomethylated polystyrene resin (0.549 g) of a loading capacity of 0.91 mmolg^−1^ [39].

### 3.2. Characterisation Studies

#### 3.2.1. Analogue **1.61**

**Figure 3:**
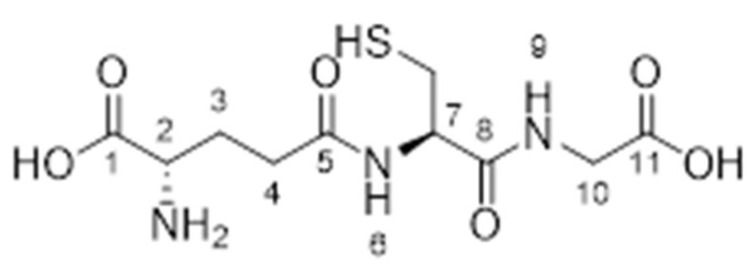
The chemical structure of Analogue **1.61**.

Compound **1.61** was characterised using multiple analytical techniques. Electrospray ionisation mass spectrometry (ESI-MS) was used to confirm the molecular ion peak [M + H^+^] at *m*/*z* 308.0 with a crude yield of 54% and a purity of 94% (purified to >95% before use). Fourier-transform infrared (FTIR) spectroscopy showed characteristic bands at 2558.3 cm^−1^ (S-H thiol stretch), 1724.2 cm^−1^ (C=O carboxylic acid stretch), and 1644.1 cm^−1^ (amide stretch). Proton nuclear magnetic resonance (^1^H NMR, 400 MHz, D_2_O) revealed chemical shifts at δH 2.21 (q, 2H, H-3), 2.59 (td, 2H, H-4), 2.98 (dd, 2H, CH_2_S), 3.86 (t, 1H, H-2), 4.02 (s, 2H, H-10), and 4.60 (t, 1H, H-7). Carbon nuclear magnetic resonance (^13^C NMR, 100 MHz, D_2_O) identified signals at δC 25.33 (CH_2_S), 25.97 (CH_2_, C-3), 31.16 (CH_2_, C-4), 41.46 (CH_2_, C-10), 53.77 (CH, C-2), 55.61 (CH, C-7), 172.44 (C=O, C-8), 173.52 (C=O, C-5), and 174.88 (C=O, C-1 and C-11). The predicted LogP values were −4.971 (Molinspiration) and −3.23 (Cambridgesoft), with predicted pKa values of 1.94, 3.74, and 9.22 (Marvin), as well as 2.118, 3.632, and 9.369 (Cambridgesoft). These findings comprehensively confirm the structure and properties of Compound 1.61, as shown in Figure 3.

#### 3.2.2. Analogue **1.62**

**Figure 4:**
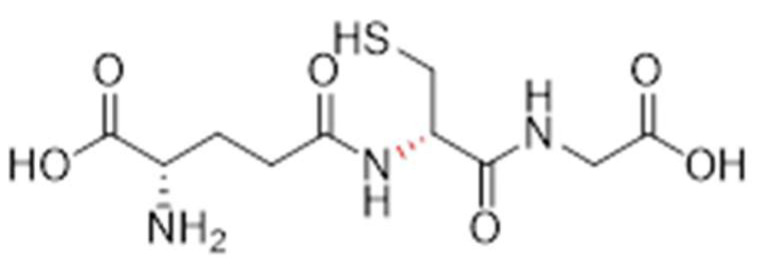
The chemical structure of Analogue **1.62** (modifications in red).

Compound **1.62** was extensively characterised, and its structural and chemical properties were confirmed. The molecular ion peak [M + H^+^] observed at *m*/*z* 308.1 in electrospray ionisation mass spectrometry (ESI-MS) aligned with the expected mass. The synthesis achieved a high yield of 94% with a purity of 81% (purified to >95% before use). The Fourier-transform infrared (FTIR) spectroscopy exhibited characteristic absorption bands, including 2562.4 cm^−1^ for the S-H thiol stretch, 1723.6 cm^−1^ for the C=O carboxylic acid stretch, and 1644.4 cm^−1^ for the amide stretch. Proton nuclear magnetic resonance (^1^H NMR, 400 MHz, D_2_O) revealed signals at δH 2.21 (q, 2H, H-3), 2.59 (td, 2H, H-4), 2.98 (dd, 2H, CH_2_S), 3.86 (t, 1H, H-2), 4.02 (s, 2H, H-10), and 4.60 (t, 1H, H-7). Carbon nuclear magnetic resonance (^13^C NMR, 100 MHz, D_2_O) showed resonances at δC 25.33 (CH_2_S), 25.97 (CH_2_, C-3), 31.16 (CH_2_, C-4), 41.46 (CH_2_, C-10), 53.77 (CH, C-2), 55.61 (CH, C-7), 172.44 (C=O, C-8), 173.52 (C=O, C-5), and 174.88 (C=O, C-1 and C-11). The predicted LogP values were calculated as −4.971 (Molinspiration) and −3.23 (Cambridgesoft), while the predicted pKa values were 1.94, 3.74, and 9.22 (Marvin), as well as 2.118, 3.632, and 9.369 (Cambridgesoft). This comprehensive analysis confirms the successful synthesis and characterisation of Compound 1.62, as shown in Figure 4.

#### 3.2.3. Analogue **1.63**

**Figure 5:**
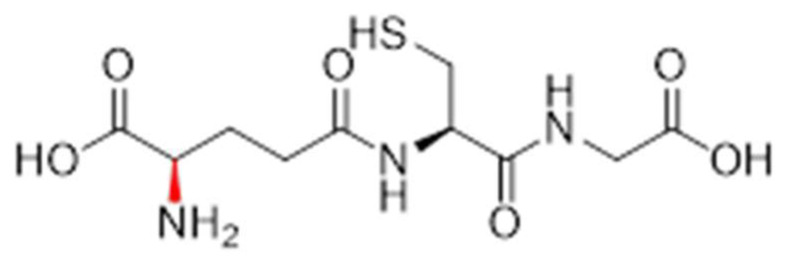
The chemical structure of Analogue **1.63** (modifications in red).

The chemical Compound **1.63** was successfully characterised, providing insights into its structure and properties. Electrospray ionisation mass spectrometry (ESI-MS) was used to identify a protonated molecular ion peak at *m*/*z* 308.2, which was found to be consistent with the expected mass. The synthesis process achieved an 87% yield and a purity of 81% (purified to >95% before use). Analysis using Fourier-transform infrared (FTIR) spectroscopy revealed characteristic peaks at 2559.2 cm^−1^ for the S-H thiol stretch, 1726.7 cm^−1^ for the C=O carboxylic acid stretch, and 1644.4 cm^−1^ for the amide group. The proton nuclear magnetic resonance (^1^H NMR, 400 MHz, D_2_O) spectra exhibited signals at δH 2.21 (quartet, 2H, H-3), 2.59 (triplet of doublets, 2H, H-4), 2.98 (doublet of doublets, 2H, CH_2_S), 3.86 (triplet, 1H, H-2), 4.02 (singlet, 2H, H-10), and 4.60 (triplet, 1H, H-7). The carbon nuclear magnetic resonance (^13^C NMR, 100 MHz, D_2_O) signals were observed at δC 25.33 (CH_2_S), 25.97 (CH_2_, C-3), 31.16 (CH_2_, C-4), 41.46 (CH_2_, C-10), 53.77 (CH, C-2), 55.61 (CH, C-7), 172.44 (C=O, C-8), 173.52 (C=O, C-5), and 174.88 (C=O, C-1 and C-11). Computational predictions estimated the LogP values as −4.971 (Molinspiration) and −3.23 (Cambridgesoft). The predicted pKa values were 1.94, 3.74, and 9.22 (Marvin), as well as 2.118, 3.632, and 9.369 (Cambridgesoft). This characterisation confirmed the successful synthesis and detailed evaluation of Compound 1.63, as shown in Figure 5.

#### 3.2.4. Analogue **1.64**

**Figure 6:**
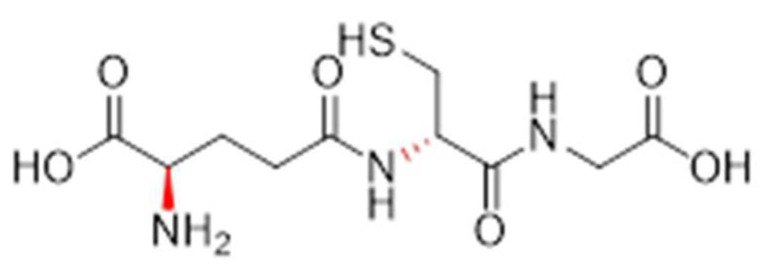
The chemical structure of Analogue **1.64** (modifications in red).

Compound 1.64 was characterised using various analytical techniques. ESI-MS analysis revealed a [M + H^+^] ion at *m*/*z* 308.2, confirming its molecular weight. The synthesis achieved a yield of 89% with a purity of 86% (purified to >95% before use). FTIR spectra showed characteristic absorption bands at 2562.4 cm^−1^ (S-H thiol stretch), 1726.7 cm^−1^ (C=O carboxylic acid stretch), and 1644.4 cm^−1^ (amide stretch). In the ^1^H NMR (400 MHz, D_2_O) spectrum, signals appeared at δH = 2.21 (q, 2H, H-3), 2.59 (td, 2H, H-4), 2.98 (dd, 2H, CH_2_S), 3.86 (t, 1H, H-2), 4.02 (s, 2H, H-10), and 4.60 (t, 1H, H-7). The ^13^C NMR (100 MHz, D_2_O) spectrum showed resonances at δC = 25.33 (CH_2_S), 25.97 (CH_2_, C-3), 31.16 (CH_2_, C-4), 41.46 (CH_2_, C-10), 53.77 (CH, C-2), 55.61 (CH, C-7), 172.44 (C=O, C-8), 173.52 (C=O, C-5), and 174.88 (C=O, C-1 and C-11). The predicted LogP values were −4.971 (Molinspiration) and −3.23 (Cambridgesoft), with pKa values estimated at 1.94, 3.74, and 9.22 (Marvin), as well as 2.118, 3.632, and 9.369 (Cambridgesoft). These findings confirm the successful synthesis and detailed structural characterisation of Compound **1.64**, as shown in Figure 6.

#### 3.2.5. Analogue **1.70**

**Figure 7:**
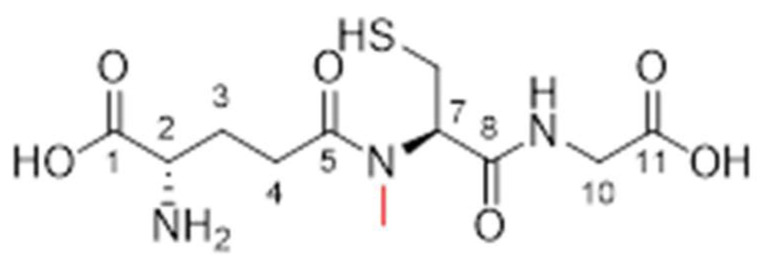
The chemical structure of Analogue **1.70** (modifications in red).

The analysis of Compound **1.70** yielded the following results: ESI-MS was used to confirm a [M + H^+^] ion at *m*/*z* 321.0, which is indicative of the expected molecular mass. The compound was obtained with a yield of 23% and a purity of 79% (purified to >95% before use). FTIR data were not available for this compound. The ^1^H NMR (400 MHz, D_2_O) analysis exhibited chemical shifts at δH = 2.24 (multiplet, 2H, H-4), 2.47 (multiplet, 1H, H-3), 2.58 (multiplet, 1H, H-3), 2.79 (singlet, 3H, NCH_3_), 3.18 (doublet of doublets, 2H, CH_2_S), 4.07 (singlet, 2H, H-10), 4.23 (triplet, 1H, H-2), and 4.43 (quartet, 1H, H-7). The ^13^C NMR (100 MHz, D_2_O) revealed resonances at δC = 23.75 (CH_2_S), 24.30 (CH_2_, C-3), 29.26 (CH_2_, C-4), 31.52 (NHCH_3_), 41.33 (CH_2_, C-10), 56.09 (CH, C-2), 62.06 (CH, C-7), 167.58 (C=O, C-5 and C-8), and 172.86 (C=O, C-1 and C-11). These data confirm the successful synthesis and structural integrity of Compound 1.70, as shown in Figure 7.

### 3.3. Biological Screening

#### 3.3.1. Stability

The GSH showed much higher stability under acidic conditions when compared to basic conditions, which is expected for peptides, as shown in Table 3 [40]. The degradation studies, therefore, were designed to use acidic conditions as degradation stopping agents, as well as storage methods. The t½ of 46.3 h for the HBSS buffer (pH 7.4) also indicated the viable timeframes of the subsequent experiments that require physiological conditions, such as cell culture studies.

#### 3.3.2. Enzymatic and Cell Degradation

The screening for potential degradation enzymes against GSH was conducted using GGT as a positive control. All of the enzymes and mixtures were screened, and none showed similar effects on GSH degradation compared to GGT, even those at much higher concentrations than that of the GGT used. Using GGT as a positive control, the enzymes (including trypsin, pepsin, chymotrypsin, aminopeptidase, jejunum luminal contents, and jejunum mucosal contents) showed insignificant GSH degradation when compared to GGT, as shown in Table 4. These enzymes were, therefore, not used for further screening experiments with the novel synthesised compounds.

#### 3.3.3. γ-Glutamyl Transferase Degradation

The D-amino acids Analogues **1.62**, **1.63**, and **1.64** showed less than half the rate of GGT degradation, indicating that GGT does not distinguish between D- and L-glutamic acid on the N-terminal (as illustrated in Table 5). This shows that the use of unnatural stereochemistry in GSH is unlikely to prevent the substantial first-pass metabolism by GGT in the liver for orally administered GSH, and it is also unlikely to aid in improving its oral bioavailability [31].

Although N-methylation modification at the glycine position (Analogue **1.65**) did not show a decrease in degradation in the presence of GGT, the N-methylation of the cysteine or glutamic acid were able to substantially decrease degradation nine-fold. This indicates that the unnatural stereochemistry on the N-terminal and cysteine–glutamic acid peptide bond can interfere with GGT activity [32].

#### 3.3.4. Glutathione S-Transferase Efficacy

As shown in Table 6, qualitative analysis by GST assay showed that the D-amino acid Analogue **1.64** with both D-cysteine and D-glutamic acid modifications retained the detoxification effectiveness and was still able to interact with the GST enzyme. In contrast, the D-amino acid Analogues **1.62** and **1.63** with D-cysteine or D-glutamic acid modifications lost detoxification efficacy from their interactions with GST. As altering the stereochemistry of either the amino acids of GSH diminished activity but not both, this indicates that the GST enzyme may only be stereospecific towards compounds with different orientations within the same compound. Although Compounds **1.62** and **1.63** lost the efficacy for a major detoxification process of GSH by being unable to interact with the GST enzyme, this does not indicate a loss for all the detoxification pathways.

The N-modification on the glycine (**1.65**) and glutamic acid (**1.71**) showed a loss of activity for the GST enzyme, whereas the modified cysteine analogue (**1.70**) did not. Although Compounds **1.65** and **1.71** lost efficacy in a major detoxification process of GSH by being unable to interact with the GST enzyme, this did not indicate a loss for all detoxification pathways.

#### 3.3.5. Glutathione Peroxidase and Glutathione Reductase Efficacy

As shown in Table 6, a qualitative analysis of the analogues showed that Compound **1.63**, with a change in D-glutamic acid stereochemistry, retained activity with GPx and GR enzymes; however, Compounds **1.62** and **1.64**, both with changes in D-cysteine stereochemistry, showed a loss of activity with GPx or GR. This suggests that either the GPx or GR enzymes specificity relies on the orientation of the cysteine residue but not the γ-glutamic acid residue. The stereochemistry of the cysteine needs to be in the L- configuration to maintain the antioxidation cycle of the GSH analogues. A loss of activity towards this major antioxidation pathway does not indicate a loss of all antioxidation properties.

All of the N-modified analogues (**1.65**, **1.70**, and **1.71**) showed a retention of activity with the GPx and GR enzymes, indicating that antioxidation activities through this pathway is not hindered by modification at the peptide bonds.

#### 3.3.6. Cytotoxicity

DMEM was unable to buffer the concentration ranges of each compound beyond 3.25 mmolL^−1^ due to the acidic nature of GSH and the synthesised compounds, which set the limit of drug concentration useable in the cell culture studies. Each compound, with the exception of **1.65**, showed no cytotoxicity up to 3.25 mmolL^−1^, and there was mostly over 100% cell viability on Caco-2, Fibroblast, and HT29-MTX-E12 cells at each tested concentration (as shown in Table 7). This indicates not only an IC50 > 3.25 mmolL^−1^, but it also indicates a cytoprotective or cytoproliferative effect of GSH and GSH-like compounds.

#### 3.3.7. UV Rescue

As shown in Table 8, the fibroblast cells treated with **1.61**, **1.62**, **1.63**, and **1.64** all showed significantly higher cell viability than the untreated groups. Although Compounds **1.62** and **1.64** lost activity for the GPx and/or GR enzymes responsible for the antioxidative effect and regeneration of the thiol group of GSH using other reductant sources, respectively, these compounds were still able to rescue the cell death of UVA irradiated cells as the neutralisation the free radicals produced by UVA irradiation could be achieved by other pathways.

Similarly, with the N-methylated analogues (**1.65**, **1.70**, and **1.71**), all of Compounds **1.65**, **1.70**, and **1.71** showed significantly higher cell viability than the untreated groups.

#### 3.3.8. In Vivo Study

**Figure 8:**
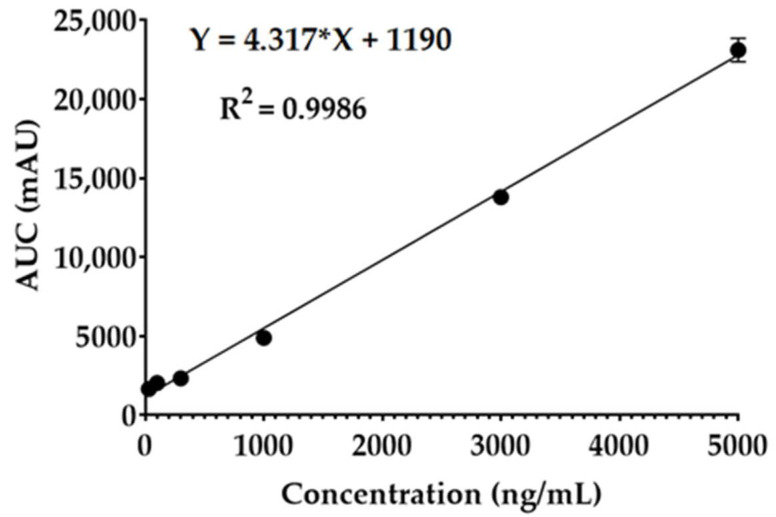
The LC–MS calibration curve of Analogue **1.70** (mean ± SD, *n* = 3).

**Figure 9:**
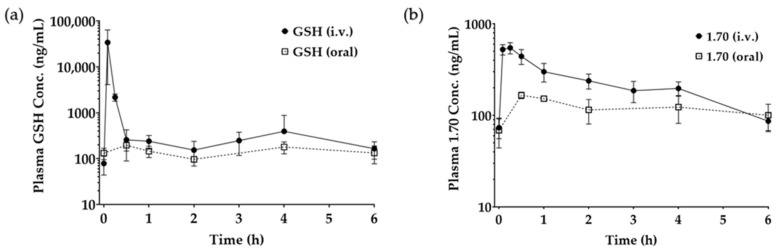
Plasma concentration of the (**a**) GSH and (**b**) Compound **1.70** in SD rats following IV injection and oral administration of drug solution (mean ± SD, *n* = 4).

Taking into consideration the results from the series of screening studies above, Compound **1.70** was selected for in vivo study as it showed retention in activity with all of the key enzymes (GST, GPx, and GR) while also displaying improved stability, as opposed to the other compounds that had lost activity with one or more key enzymes. A dedicated LC-MS method for **1.70** was successfully developed and validated for linearity with concentration ranges from 30 to 5000 ng/mL, as demonstrated in Figure 8. Based on this, the plasma concentrations of 1.70 was analysed, as shown in Figure 9 and Table 9. The plasma t½ of Compound **1.70** was measured to be 33.6 min compared to the plasma t½ of the native GSH of 2.0 min, displaying a 16.8-fold improvement. This improvement is in correspondence with our in vitro degradation data. The N-methylation of **1.70** caused decreased GGT degradation. Similar chemical modifications of other peptides have also shown that N-methylation can decrease enzymatic degradation [41]. A longer plasma half-life (t½) of Analogue 1.70 offers several therapeutic advantages over native GSH, particularly in maintaining sustained antioxidant and detoxification effects. The 16.8-fold increase in t½ (from 2.0 min to 33.6 min) significantly prolongs systemic circulation, reducing the need for frequent dosing [42]. The prolonged GSH in body is crucial for protecting against oxidative stress-related diseases [43]. The oral bioavailability of Analogue **1.70** was calculated to be 11.3 ± 1.2%, showing a 16.1-fold improvement over native GSH, which has an oral bioavailability of only 0.7 ± 0.1% [44]. In addition, an improvement in oral bioavailability also corresponded with our in vitro degradation data as the GGT degraded GSH during first pass metabolism. N-methylation also decreased the hydrophilicity of GSH, making it more suitable for transport through the intestinal epithelium. Improved oral bioavailability due to N-methylation of other peptides has also been reported [45]. The 16.1-fold increase in oral bioavailability significantly advances the potential of a non-invasive oral glutathione option. These results show similar oral bioavailability to other peptide/proteins when using analogue methods by displaying up to 10% relative oral bioavailability [46].

The variation between the three tested compounds in absolute AUC raises a concern as it may indicate experimental error in the earlier time points due to rapid degradation or distribution into tissue [47]. Although this pharmacokinetic study managed to measure the increase in plasma glutathione concentration, it has been reported that excess GSH is rapidly internalised into the liver [48]. Further investigation into tissue distribution is necessary to confirm whether the longer t½ translates into increased intracellular GSH levels in target organs such as the liver, brain, and kidneys, which are critical for its detoxifying and protective roles.

## 4. Conclusions

This study successfully demonstrated that strategic chemical modifications to glutathione (GSH) can significantly enhance its oral bioavailability, overcoming the key limitations associated with its native form. Among the synthesised analogues, Compound **1.70**, which is an N-methylated cysteine derivative, exhibited the most promising pharmacokinetic and biological properties. It demonstrated superior resistance to enzymatic degradation, enhanced cellular viability, and improved antioxidant efficacy. Notably, the in vivo pharmacokinetic evaluations in a Sprague Dawley rat model revealed a 16.8-fold increase in plasma half-life and a 16.1-fold improvement in oral bioavailability compared to native GSH. These findings underscore the potential of N-methylated GSH derivatives as viable candidates for oral administration, thereby offering a breakthrough in antioxidant therapy. Given their enhanced stability and bioavailability, these analogues hold promise for therapeutic applications in conditions linked to oxidative stress, including neurodegenerative disorders, chronic inflammation, and metabolic diseases. Future research should focus on further optimisation of these analogues, exploring formulation strategies that maximise absorption and therapeutic effectiveness. Although this study showed no cytotoxic effects of the analogues on cells at the highest concentration (3.25 mmol/L), or any visible adverse effects on rats at near the maximum equivalent human dose for GSH (1000 mg/kg), it is worth noting that the long-term effects and analysis of the effects of the analogue’s metabolites were beyond the scope of this study. As it stands, doses similar to GSH’s should be viable in human use for the analogues; however, a thorough investigation of metabolites, therapeutic efficacy, pharmacodynamics, and tissue distribution will be required for safety before clinical validation through human trials can be performed. Overall, this study paves the way for the development of next-generation GSH-based therapeutics, providing a robust foundation for addressing oxidative stress-related pathologies through an effective and bioavailable oral delivery strategy.

## Figures and Tables

**Table 1:** The chemical structure of synthesised glutathione and its analogues.

Name	Structure	Molecular Weight/gmol^−1^
**1.61**	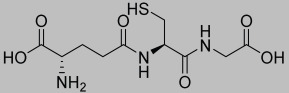	307.32
**1.62**	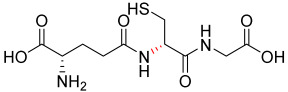	307.32
**1.63**	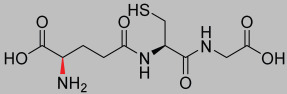	307.32
**1.64**	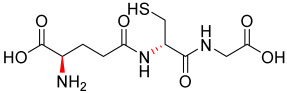	307.32
**1.65**	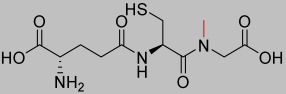	321.35
**1.70**	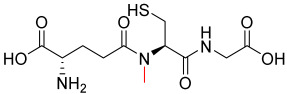	321.35
**1.71**	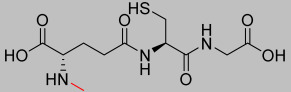	321.35

**Table 2:** The amounts of each building block used in the synthesis of **1.61**–**1.65** and **1.70**–**1.71**.

Compound	Building Block A, 3eq	Mass of A/g	Building Block B, 5eq	Mass of B/g	Building Block C, 5eq	Mass of C/g
**1.61**	Fmoc-Gly-OH	0.446	Fmoc-Cys(Trt)-OH	1.465	Fmoc-Glu-OtBu	1.064
**1.62**	Fmoc-Gly-OH	0.446	Fmoc-D-Cys(Trt)-OH	1.465	Fmoc-Glu-OtBu	1.064
**1.63**	Fmoc-Gly-OH	0.446	Fmoc-Cys(Trt)-OH	1.465	Fmoc-D-Glu-OtBu	1.064
**1.64**	Fmoc-Gly-OH	0.446	Fmoc-D-Cys(Trt)-OH	1.465	Fmoc-D-Glu-OtBu	1.064
**1.65**	Fmoc-Sar-OH	0.467	Fmoc-Cys(Trt)-OH	1.465	Fmoc-Glu-OtBu	1.064
**1.70**	Fmoc-Gly-OH	0.467	Fmoc-Cys(Trt)-OH	1.465	Fmoc-Glu-OtBu	1.064
**1.71**	Fmoc-Gly-OH	0.446	Fmoc-Cys(Trt)-OH	1.465	Fmoc-Glu-OtBu	1.064

**Table 3:** The stability of GSH under various pH conditions at 37 °C (mean ± SD, *n* = 3).

pH	GSH t_½_ (h)
**2.0**	202.7 ± 10.1
**7.4**	46.3 ± 2.3
**12.0**	2.5 ± 0.1

**Table 4:** The degradation of GSH by various degradation enzymes (mean ± SD, *n* = 3).

Enzyme	GSH t_½_ (h)	Concentration (UmL^−1^)
**Trypsin**	5.8 ± 0.3	10
**Pepsin**	31.4 ± 1.6	10
**Chymotrypsin**	4.6 ± 0.2	10
**Aminopeptidase**	18.9 ± 0.9	10
**GGT**	0.7 ± 0.03	10
**Jejunum luminal contents**	30.5 ± 1.5	N/A
**Jejunum mucosal contents**	9.4 ± 2.0	N/A

**Table 5:** The degradation of GSH and its analogues by GGT.

Name	Equation	R^2^ Value	% Degradation
**1.61**	−0.0125x + 4.1334	0.9187	100
**1.62**	−0.0107x + 4.5977	0.9911	82.3
**1.63**	−0.0041x + 1.9982	0.9814	72.7
**1.64**	−0.0034x + 1.9619	0.9478	60.2
**1.65**	−0.0054x + 1.9908	0.9956	122.2
**1.70**	−0.0004x + 2.2060	0.8911	11.7
**1.71**	−0.0004x + 2.0026	0.9121	11.7

**Table 6:** The efficacy of the novel compounds for GST compared to the efficacy of GSH towards GST.

Name	GGT Degradation < 50%	GST Activity > 50%	GT/GPx Activity > 50%
**GSH**	X	✔	✔
**1.61**	X	✔	✔
**1.62**	X	X	X
**1.63**	X	X	✔
**1.64**	X	✔	X
**1.65**	X	X	✔
**1.70**	✔	✔	✔
**1.71**	✔	X	✔

Indicate ✔: yes. X: no.

**Table 7:** The cell viability of glutathione and its analogues.

Name	IC_50_ (mmolL^−1^)
**GSH (commercial)**	>3.25
**1.61 (synthesised)**	>3.25
**1.62**	>3.25
**1.63**	>3.25
**1.64**	>3.25
**1.65**	0.984
**1.70**	>3.25
**Name**	IC_50_ (mmolL^−1^)

**Table 8:** The cell viability of glutathione and its analogues with or without UV treatment (mean ± SD, *n* = 6).

Name	% Cell Viability (no UV)	% Cell Viability (with UV)
**GSH**	100.0 ± 8.1	87.7 ± 4.5
**1.61**	74.3 ± 2.0	69.0 ± 0.6
**1.62**	111.8 ± 5.5	70.8 ± 5.7
**1.63**	137.1 ± 16.1	101.1 ± 5.2
**1.64**	127.7 ± 7.9	98.5 ± 7.1
**1.65**	58.8 ± 0.0	69.3 ± 26.6
**1.70**	107.8 ± 9.4	112.4 ± 9.7
**1.71**	79.5 ± 10.2	97.0 ± 5.5
**Control** **(no drug)**	90.6 ± 9.5	40.1 ± 4.3

**Table 9:** The area under the curve and bioavailability for the GSH Compounds **1.70** in Sprague Dawley rats following oral or IV administration of the respective compounds (mean ± SD, *n* = 4, ** *p* < 0.05).

	GSH Solution (IV)	GSH Solution (oral)	Analogue 1.70 (IV)	Analogue 1.70 (oral)
**AUC (nghmL^−1^)**	5353 ± 2480	36 ± 8	727 ± 42	82 ± 8
**Bioavailability**	100%	0.7 ± 0.1%	100%	11.3± 1.2% **

## Data Availability

The original contributions presented in this study are included in the article/Supplementary Material. Further inquiries can be directed to the corresponding author(s).

## Supplementary Materials

The following supporting information can be downloaded at: https://www.mdpi.com/article/10.3390/pharmaceutics17030385/s1, The NMR, FTIR, HPLC, ESI-MS, LC-MS and HPLC data can be found in the Supplementary Figures S1–S26.
