# Supplementary material for: Enhancing the Oral Bioavailability of Glutathione Using Innovative Analogue Approaches
Source: Pharmaceutics. 2025 Mar 18;17(3):385. doi: 10.3390/pharmaceutics17030385 (PMC11945201; doi:10.3390/pharmaceutics17030385)
Supplement: Supplementary file 1 [file pharmaceutics-17-00385-s001.zip › pharmaceutics-3480504-supplementary.pdf]

Supplementary data

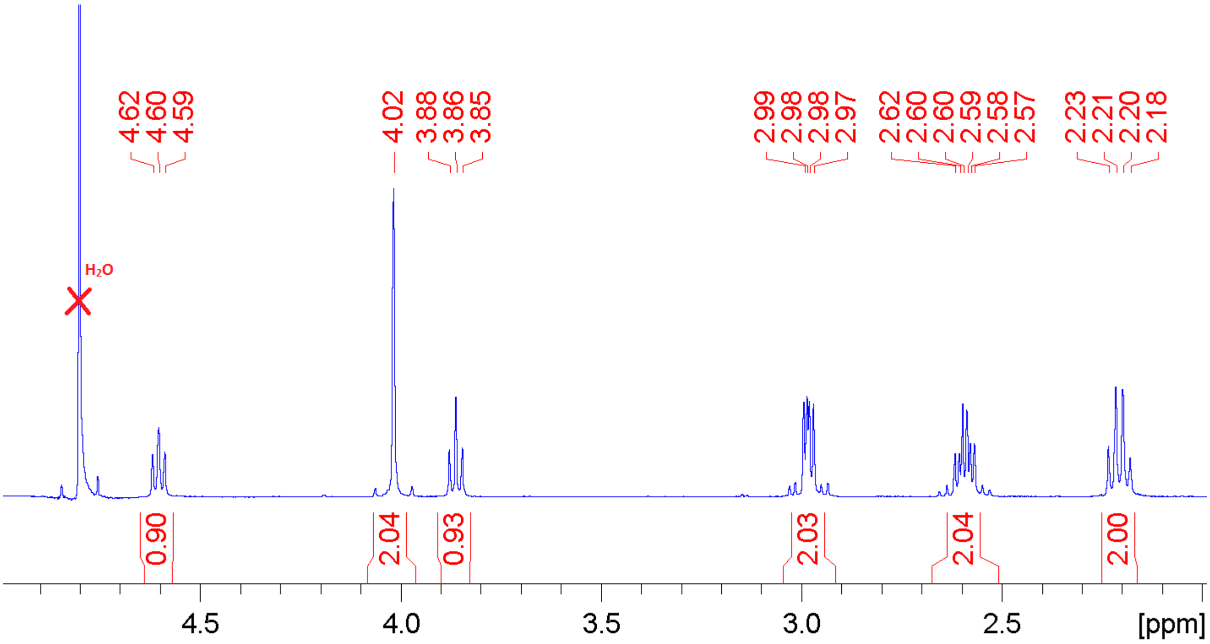

Figure S1: ^{1}H NMR spectra of crude peptide **1.61**

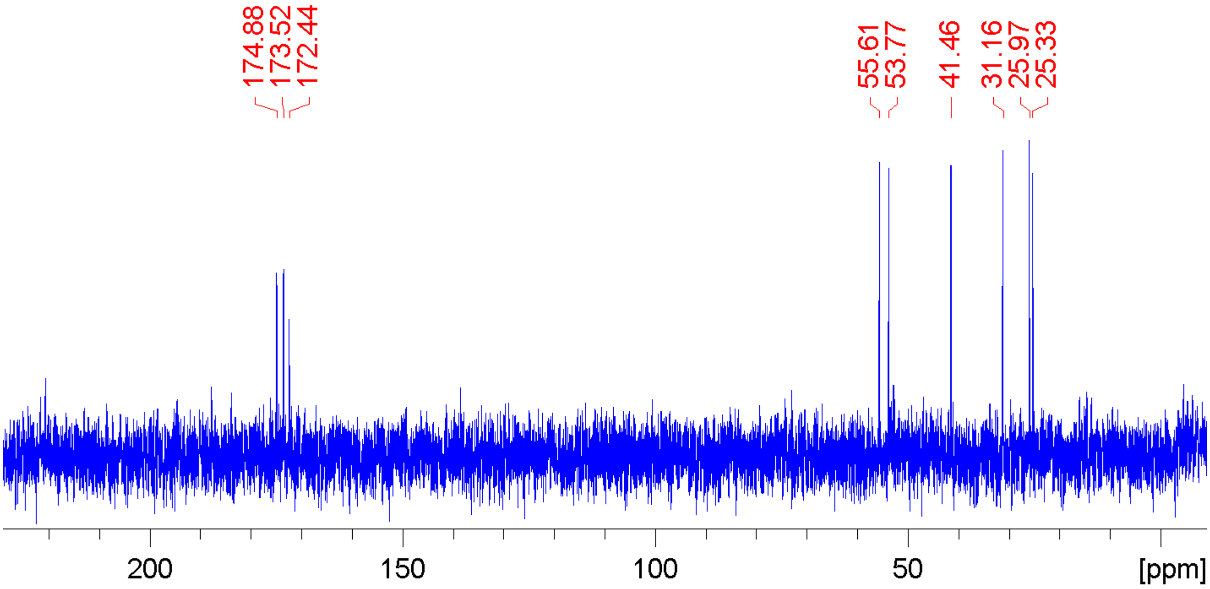

Figure S2: ^{13}C NMR spectra of crude peptide **1.61**

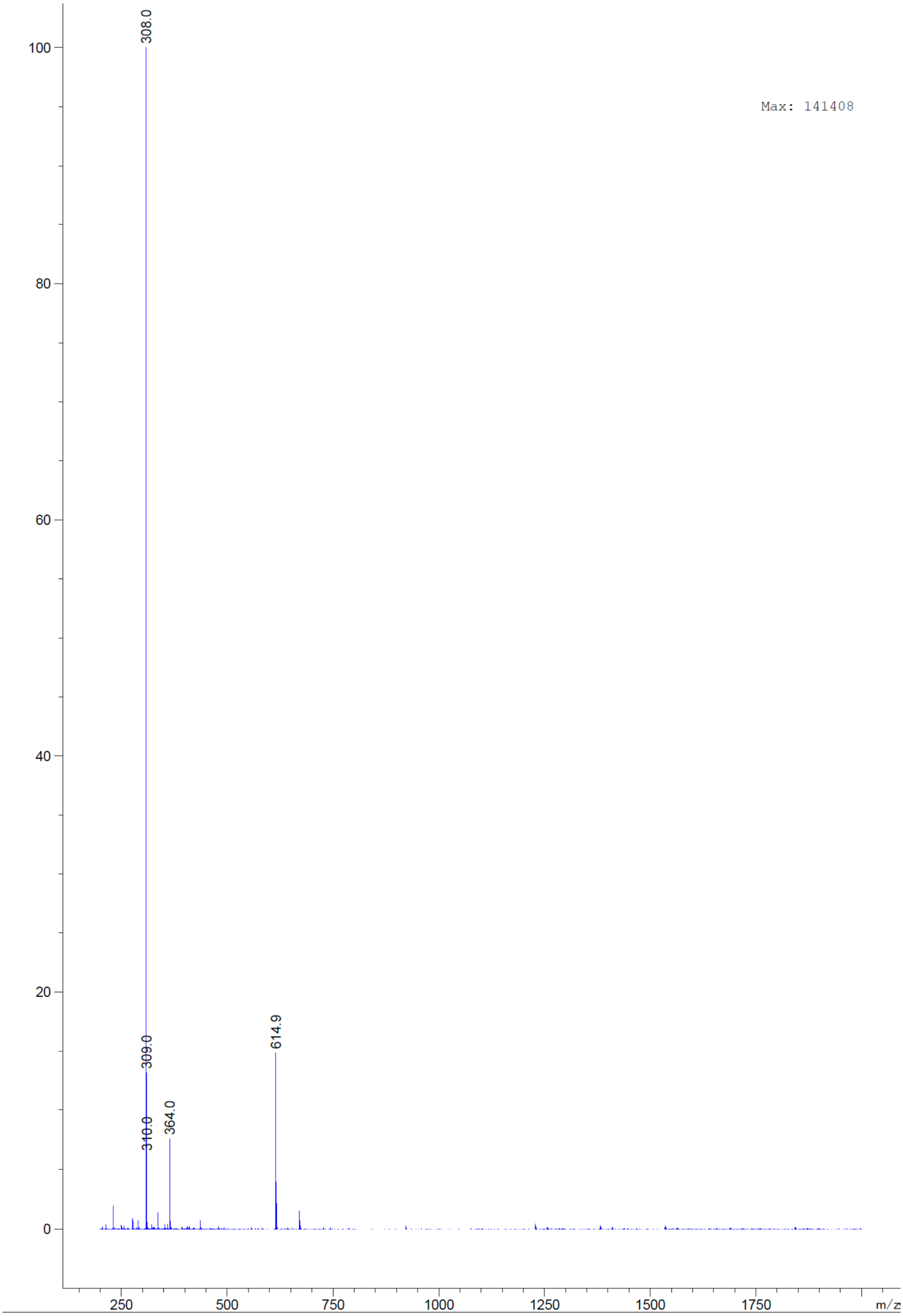

Figure S3: ESI-MS data of crude peptide **1.61**

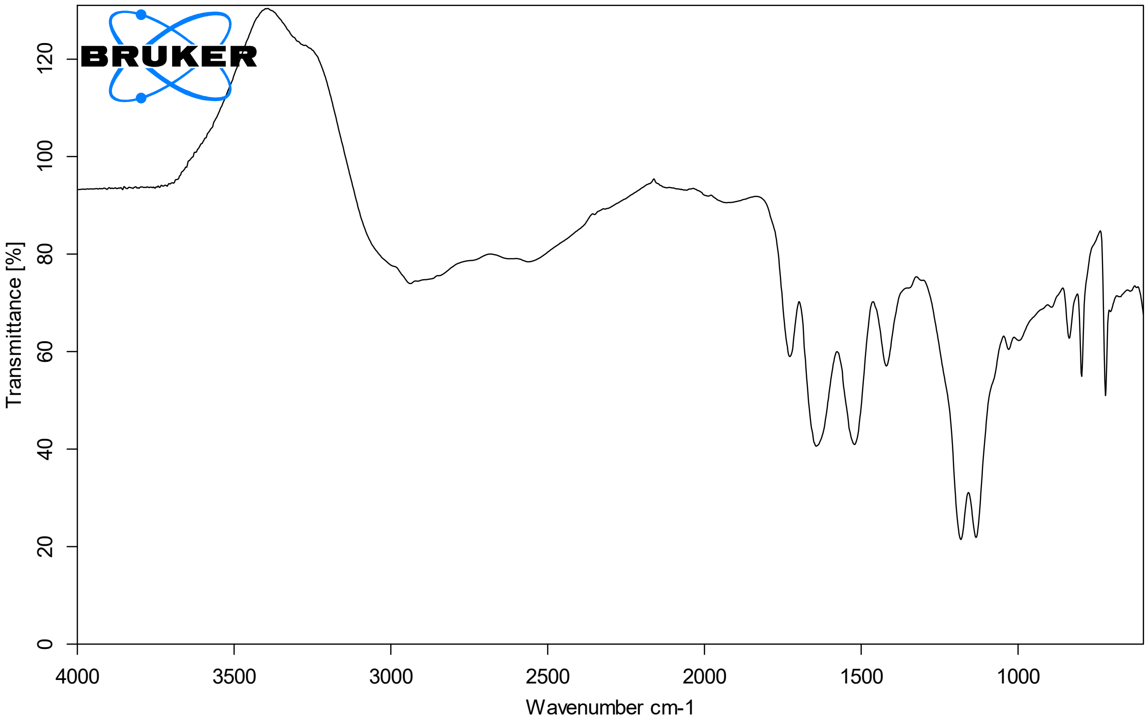

Figure S4: FTIR data of crude peptide **1.61**

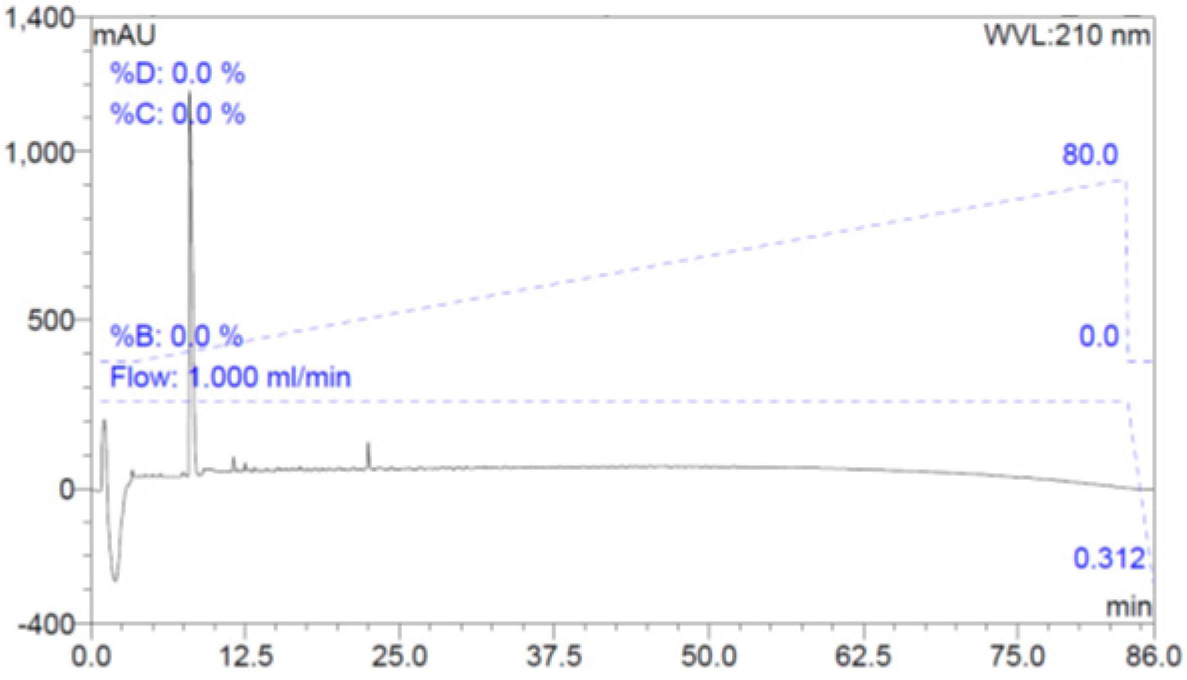

Figure S5: HPLC chromatogram of crude peptide **1.61**

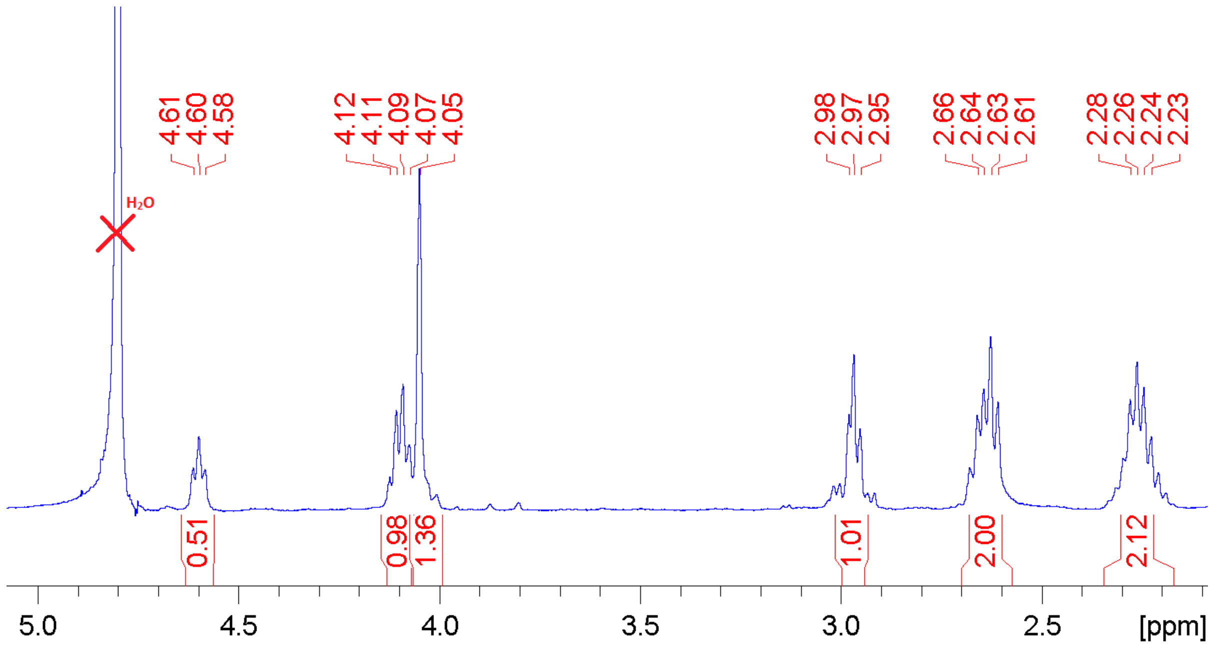

Figure S6: ^{1}H NMR spectra of crude peptide **1.62**

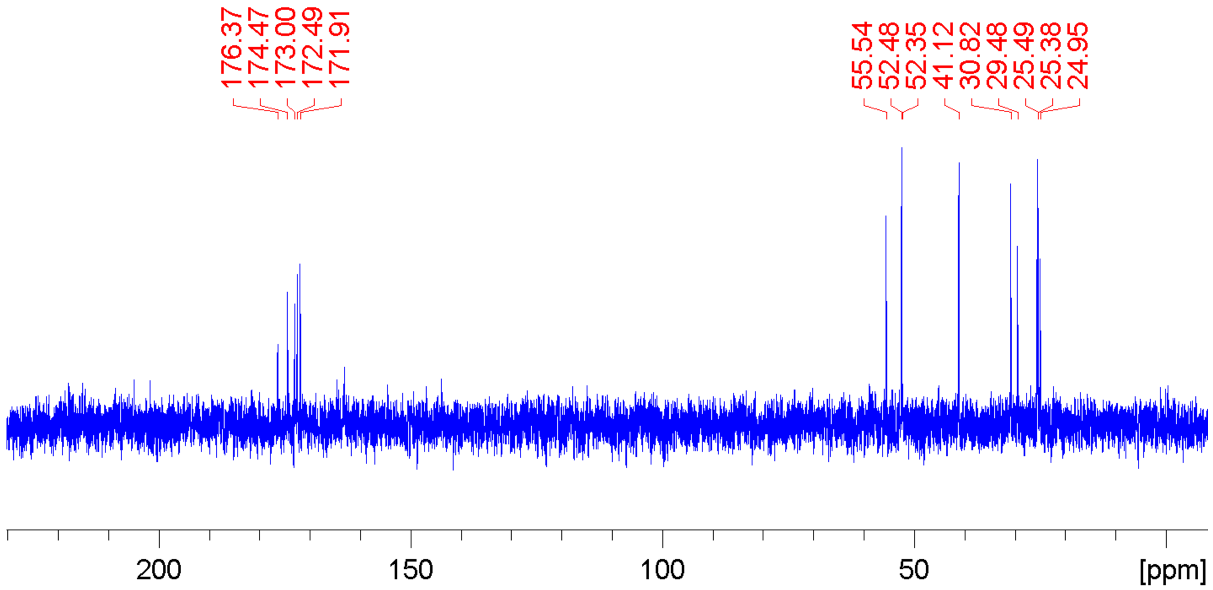

Figure S7: ^{13}C NMR spectra of crude peptide **1.62**

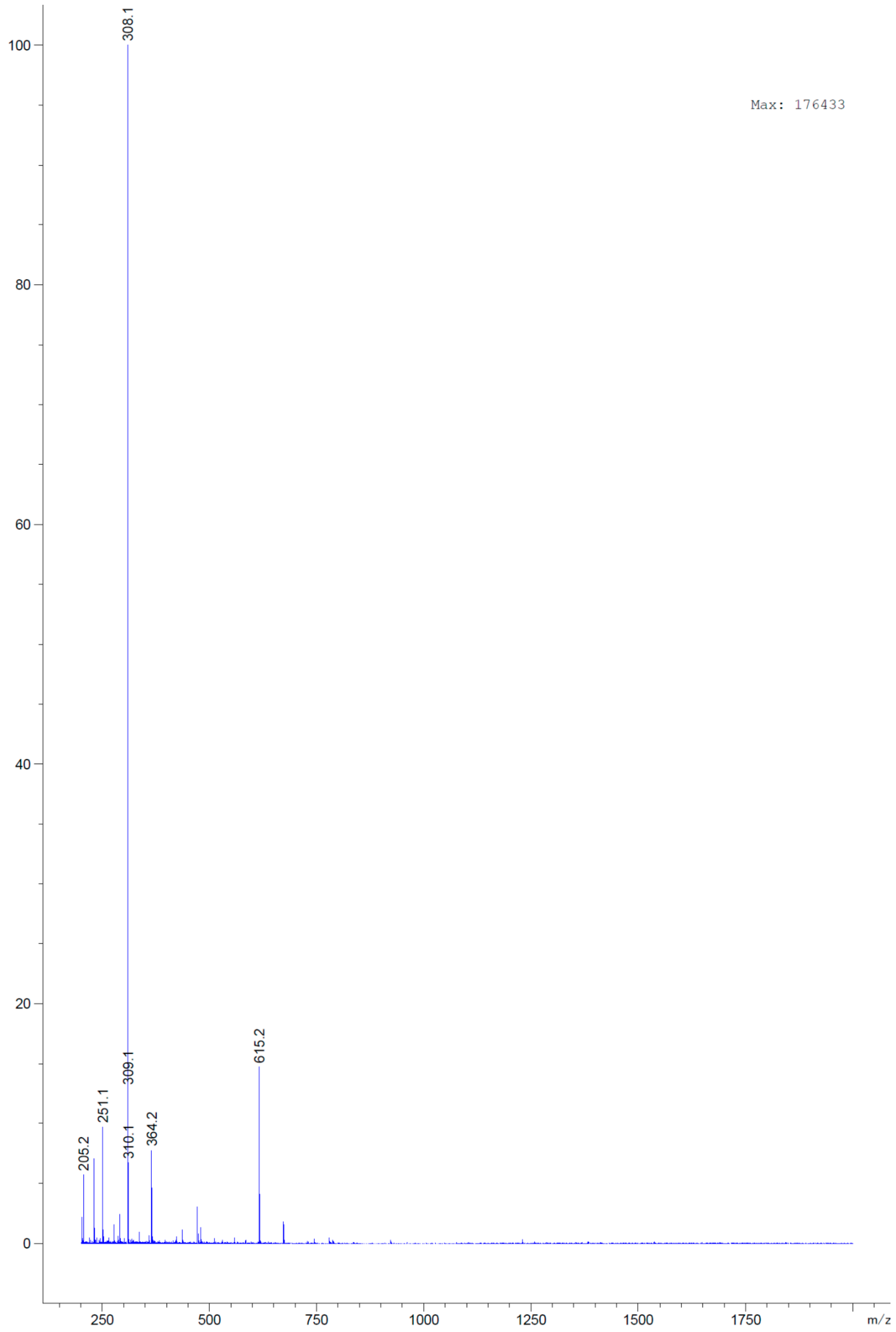

Figure S8: ESI-MS data of crude peptide **1.62**

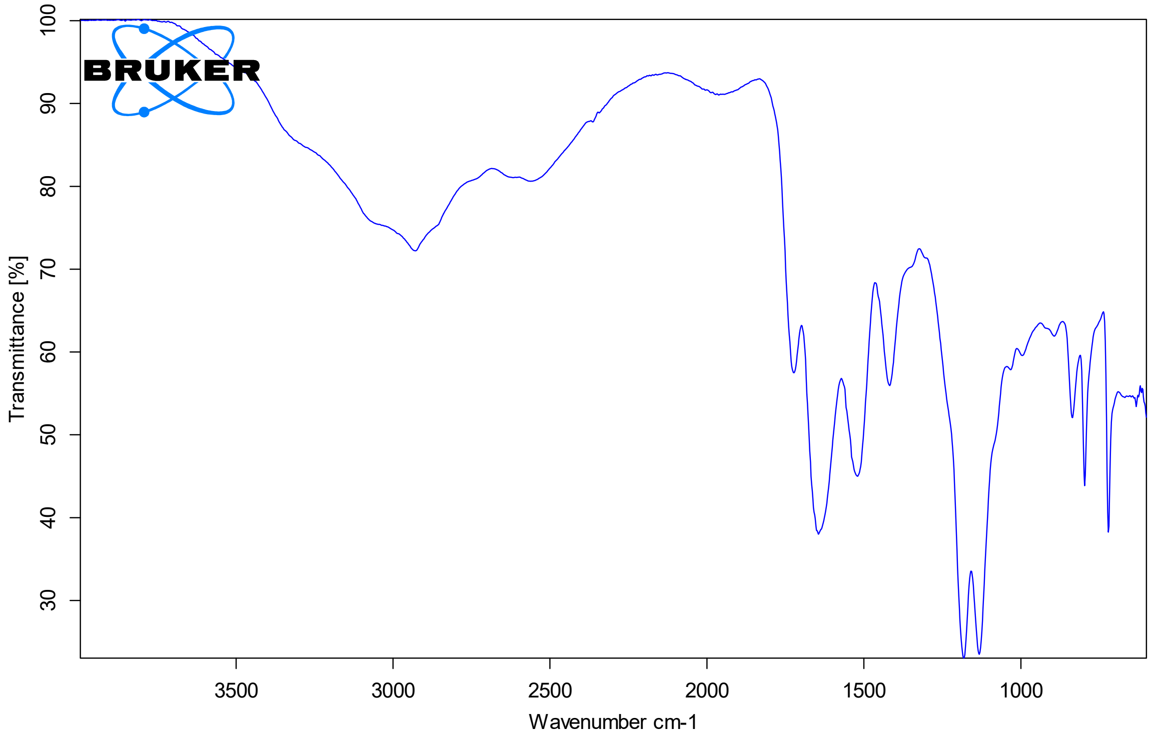

Figure S9: FTIR data of crude peptide **1.62**

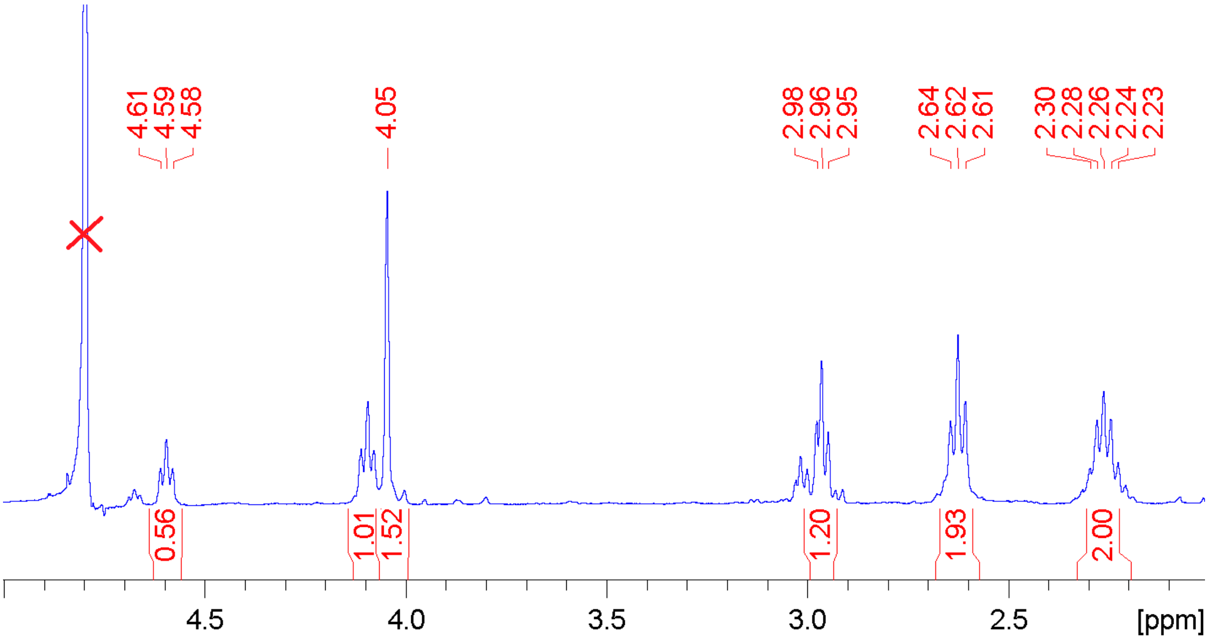

Figure S10: ^{1}H NMR spectra of crude peptide **1.63**

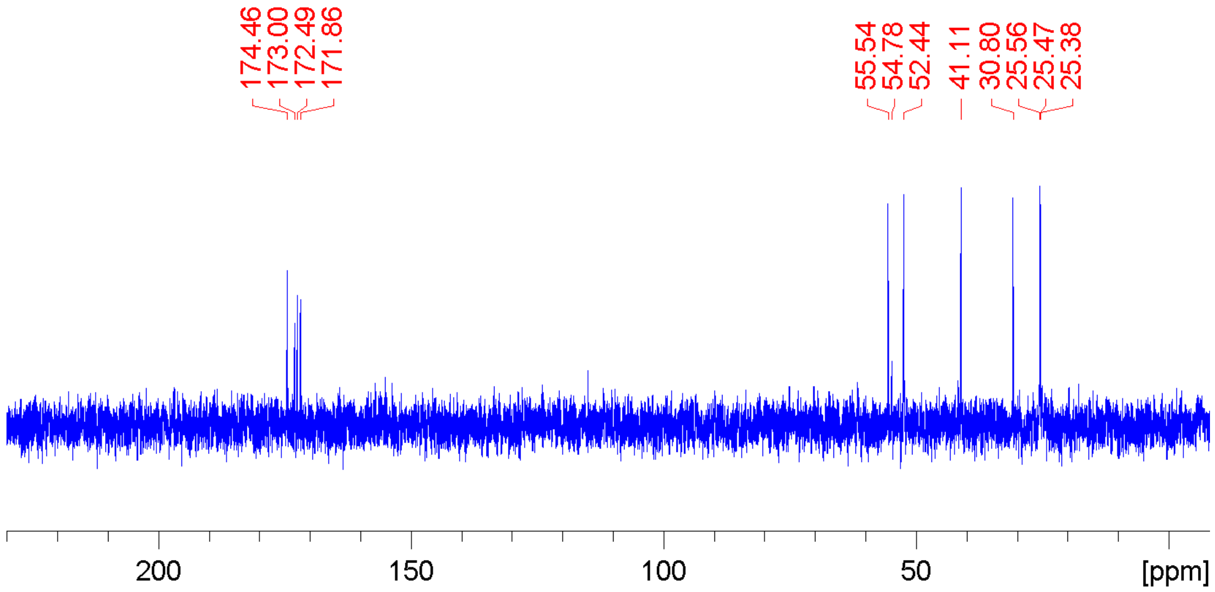

Figure S11: ^{13}C NMR spectra of crude peptide **1.63**

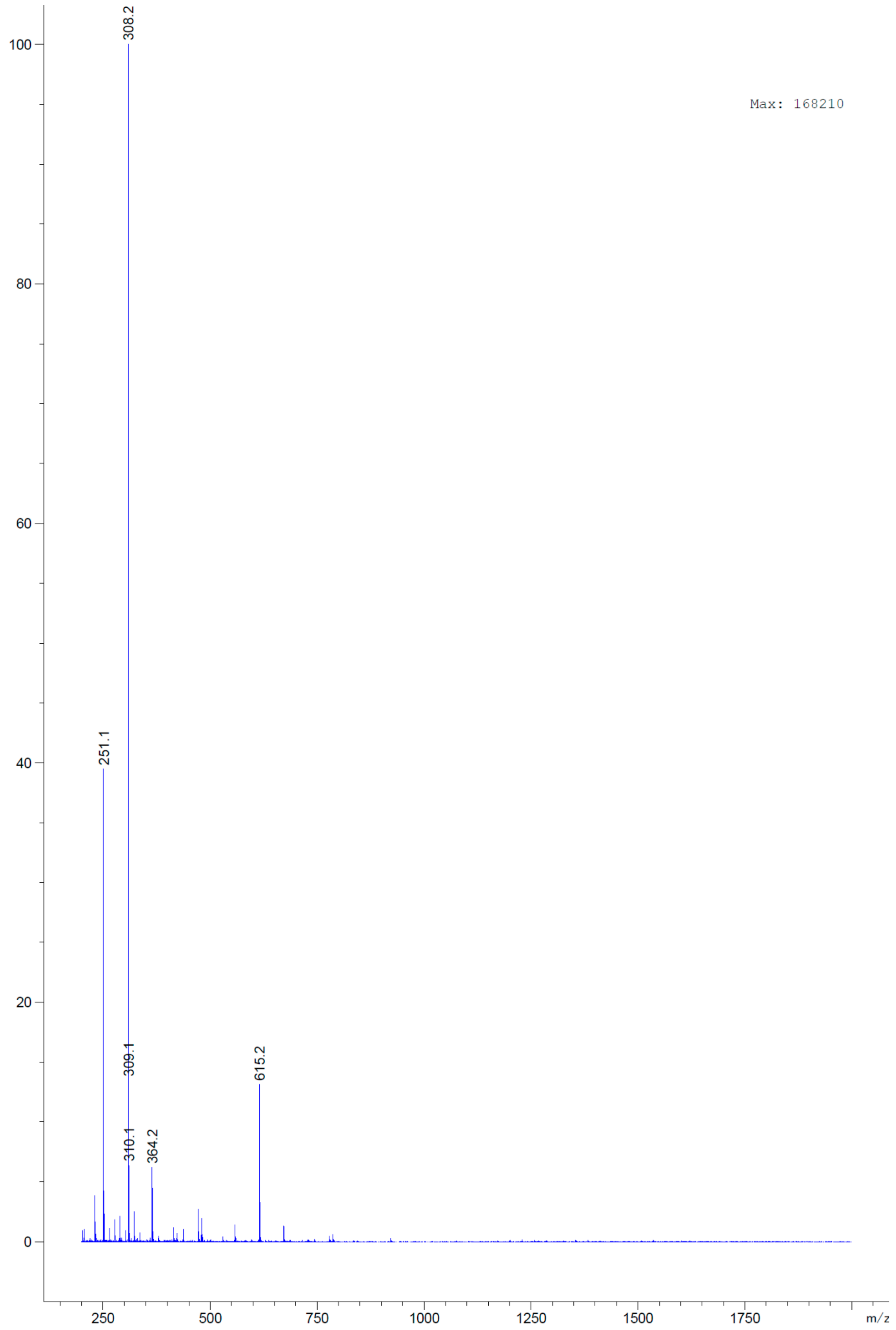

Figure S12: ESI-MS data of crude peptide **1.63**

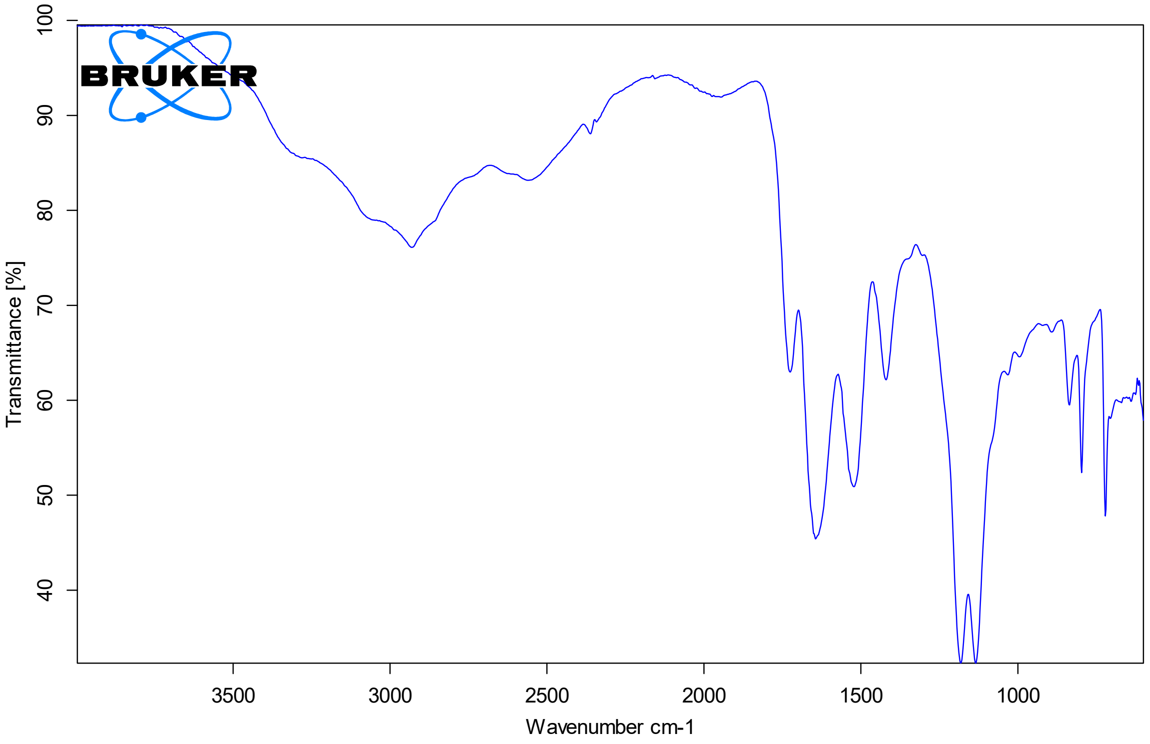

Figure S13: FTIR data of crude peptide **1.63**

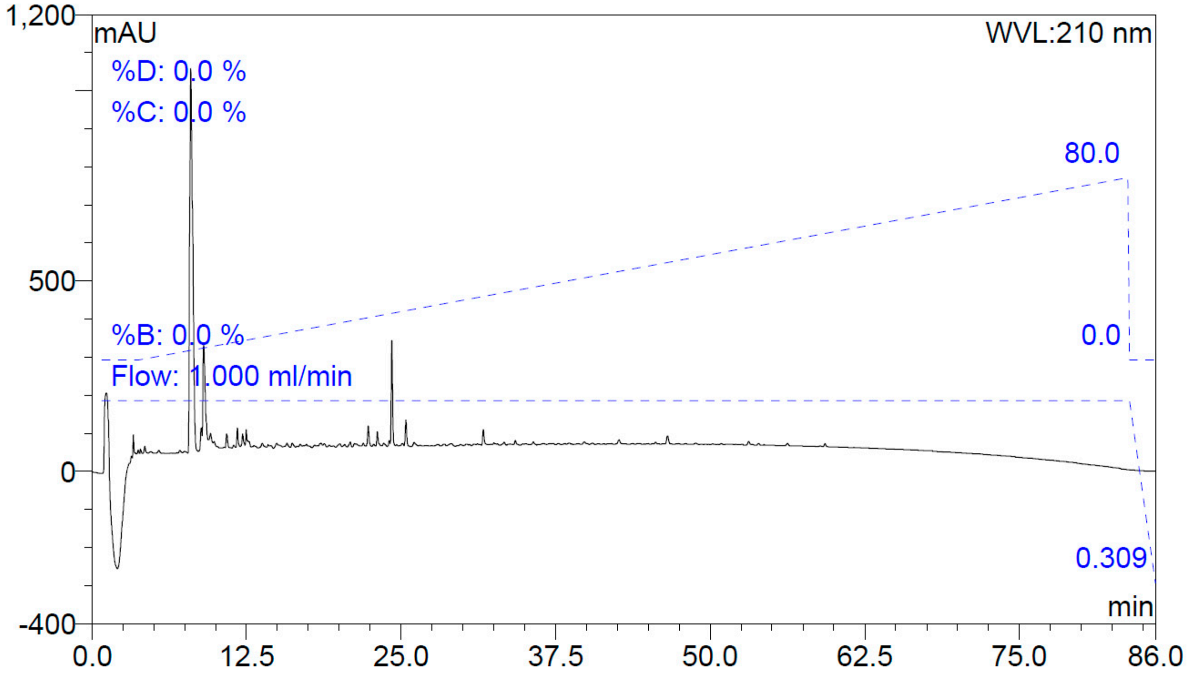

Figure S14: HPLC chromatogram of crude peptide **1.63**

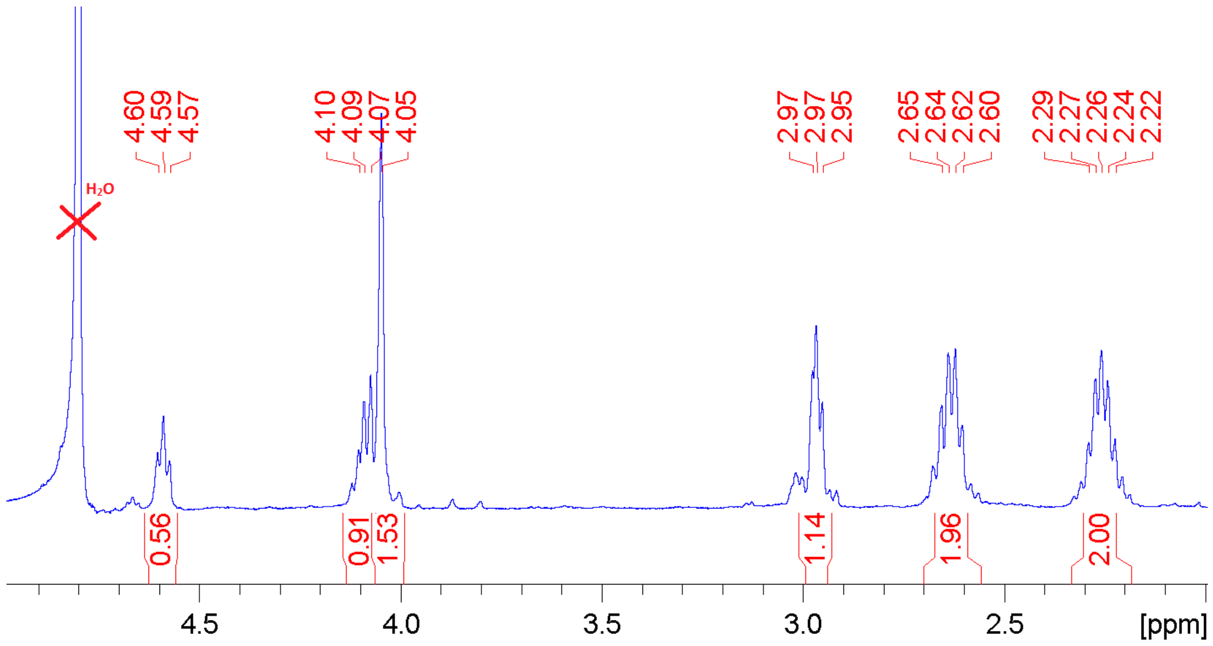

Figure S15: ^{1}H NMR spectra of crude peptide **1.64**

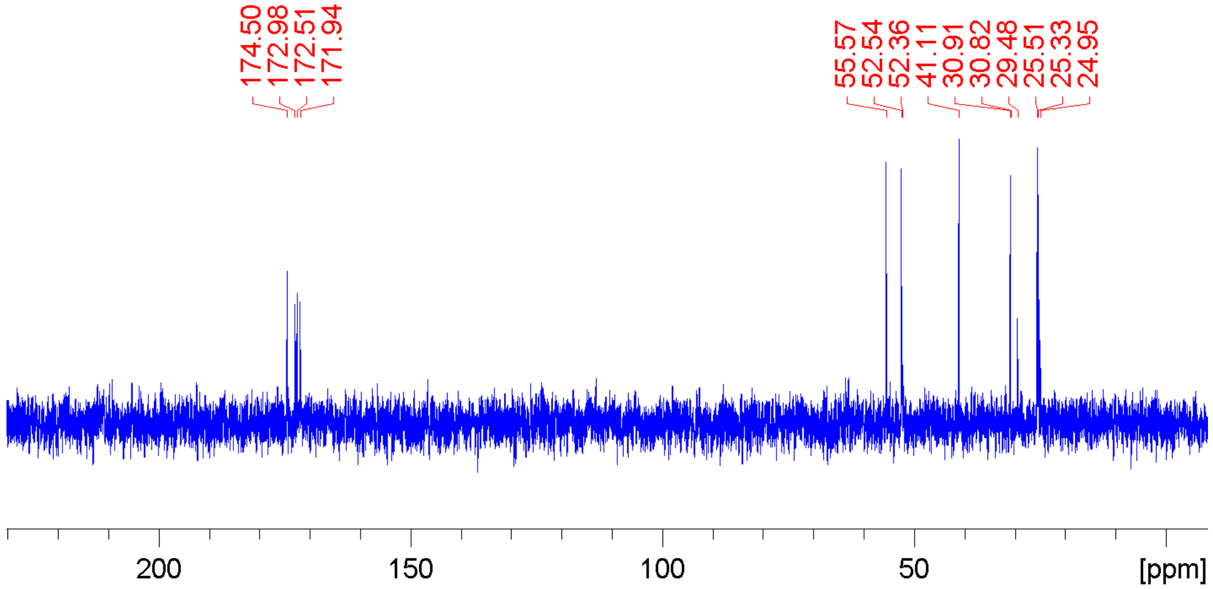

Figure S16: ^{13}C NMR spectra of crude peptide **1.64**

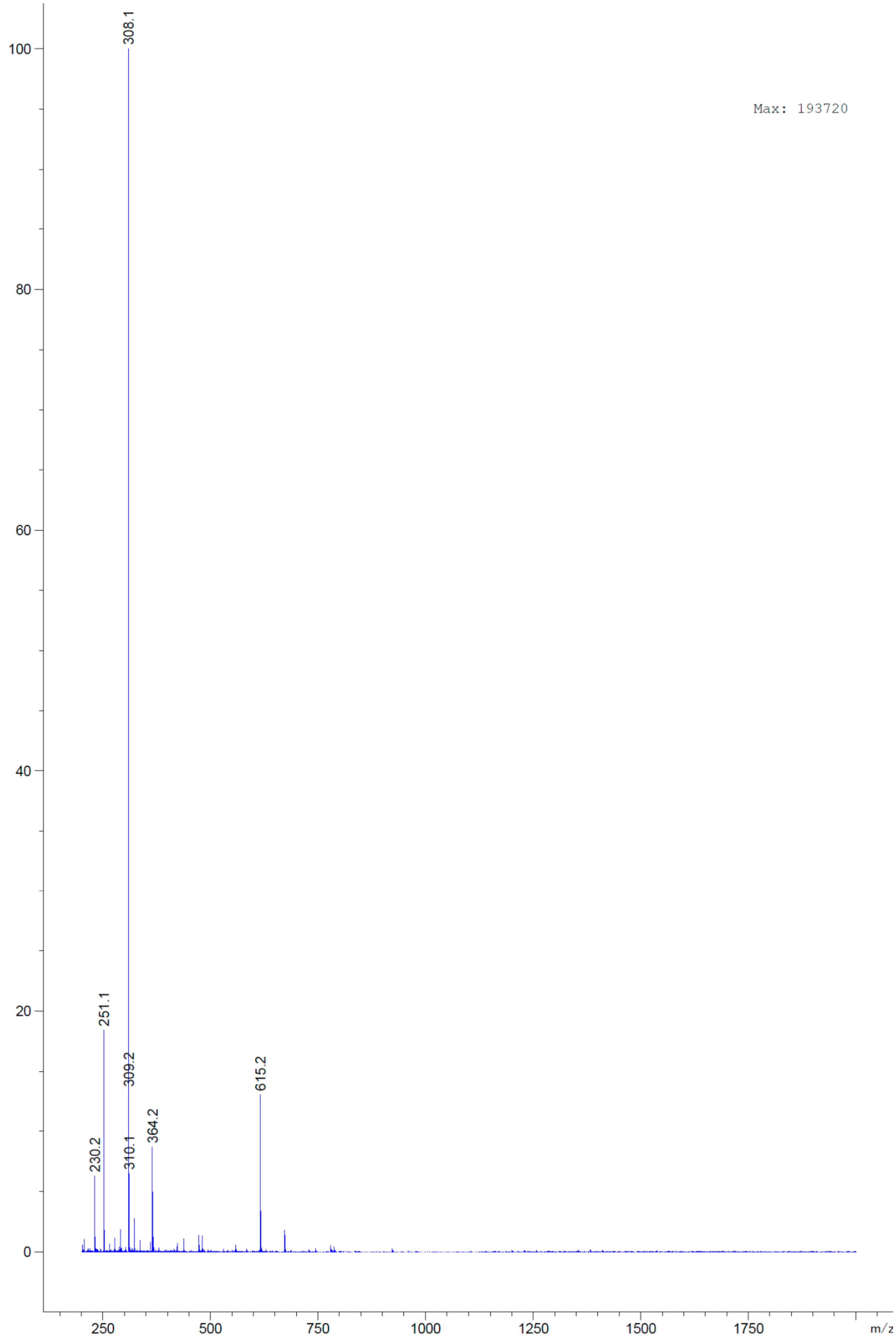

Figure S17: ESI-MS data of crude peptide **1.64**

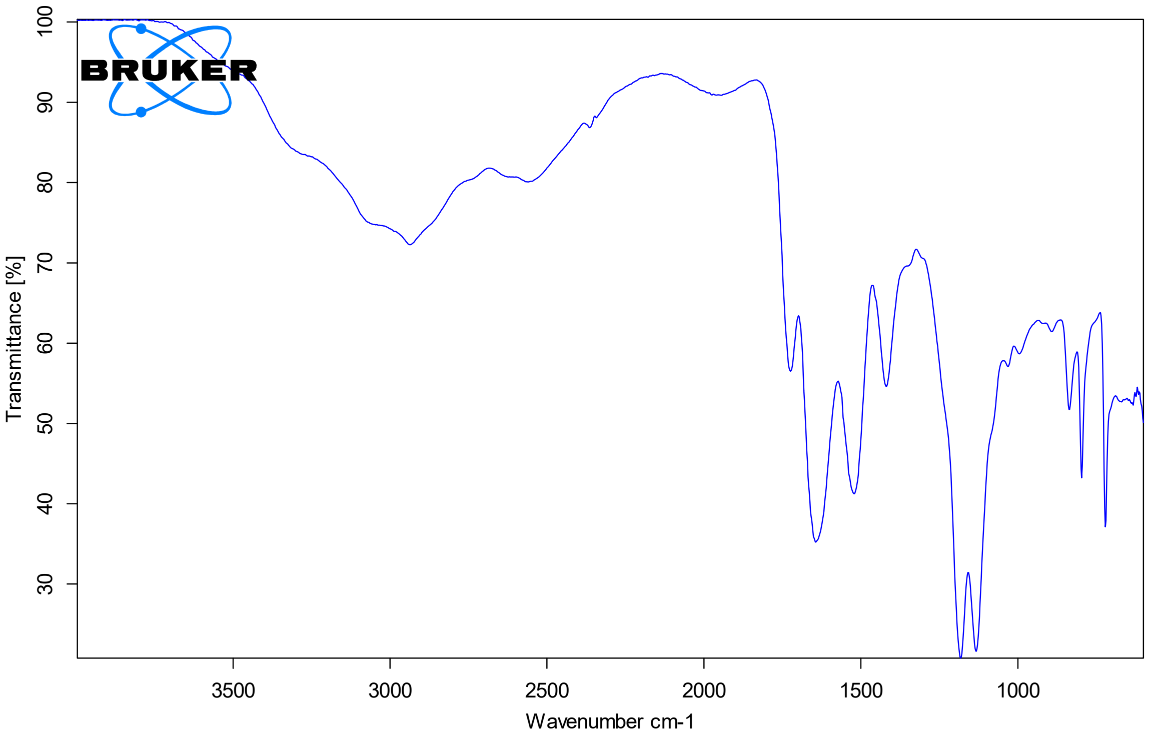

Figure S18: FTIR data of crude peptide **1.64**

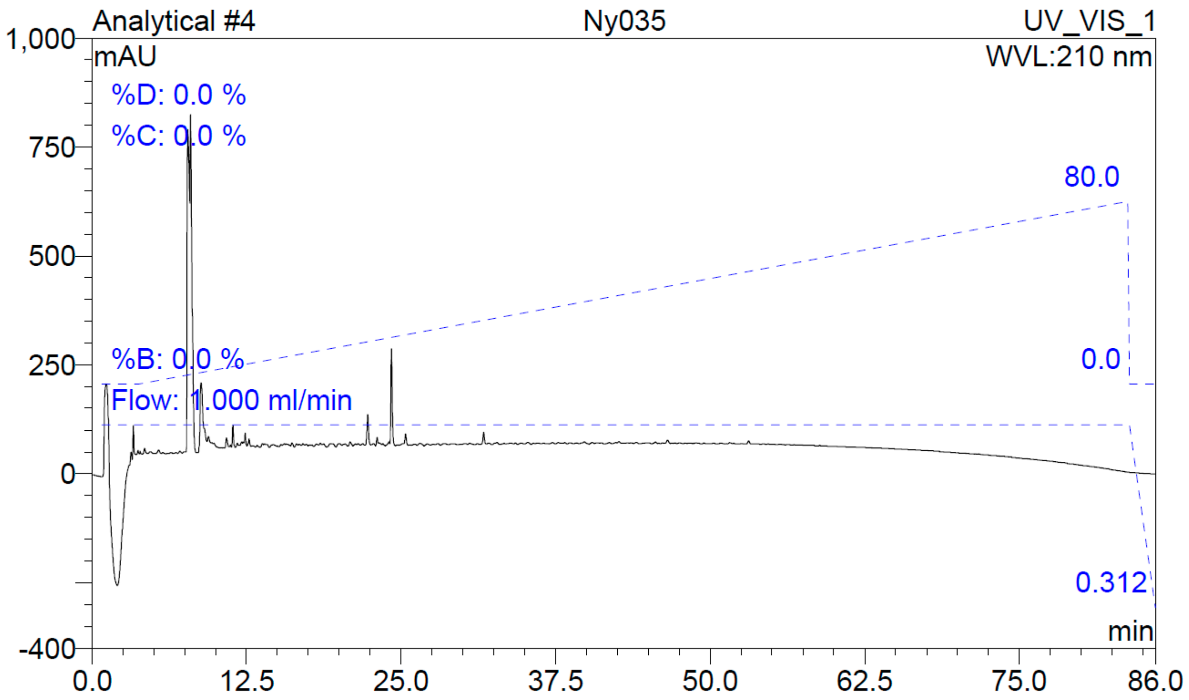

Figure S19: HPLC chromatogram of crude peptide **1.64**

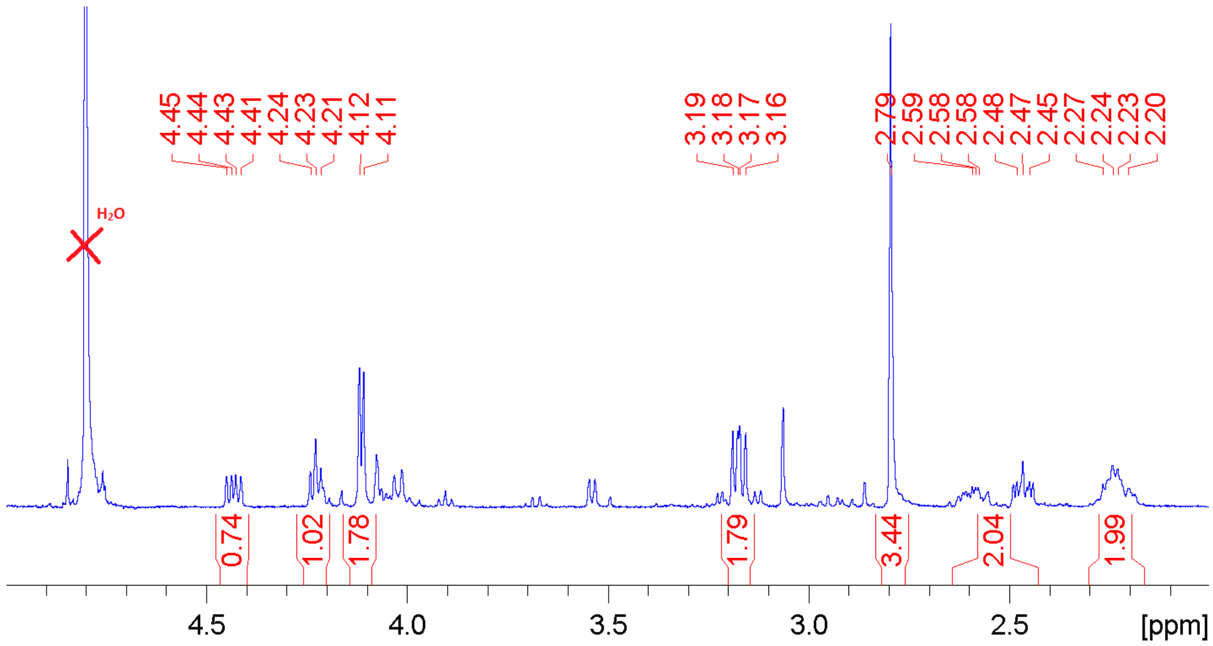

Figure S20: ^{1}H NMR spectra of crude peptide **1.70**

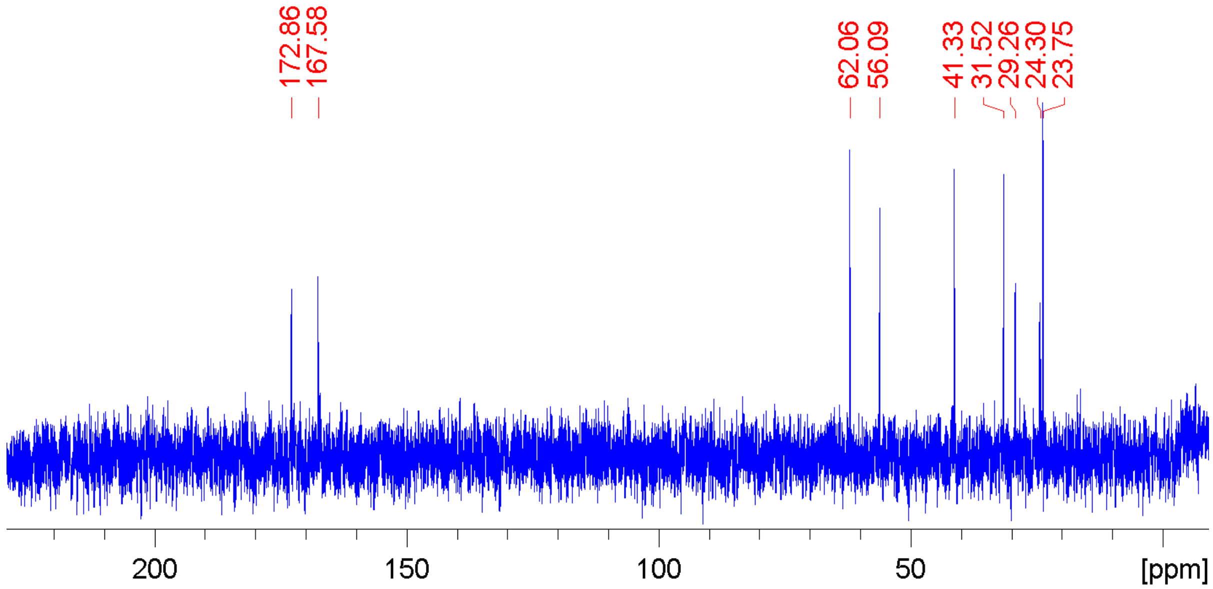

Figure S21: ^{13}C NMR spectra of crude peptide **1.70**

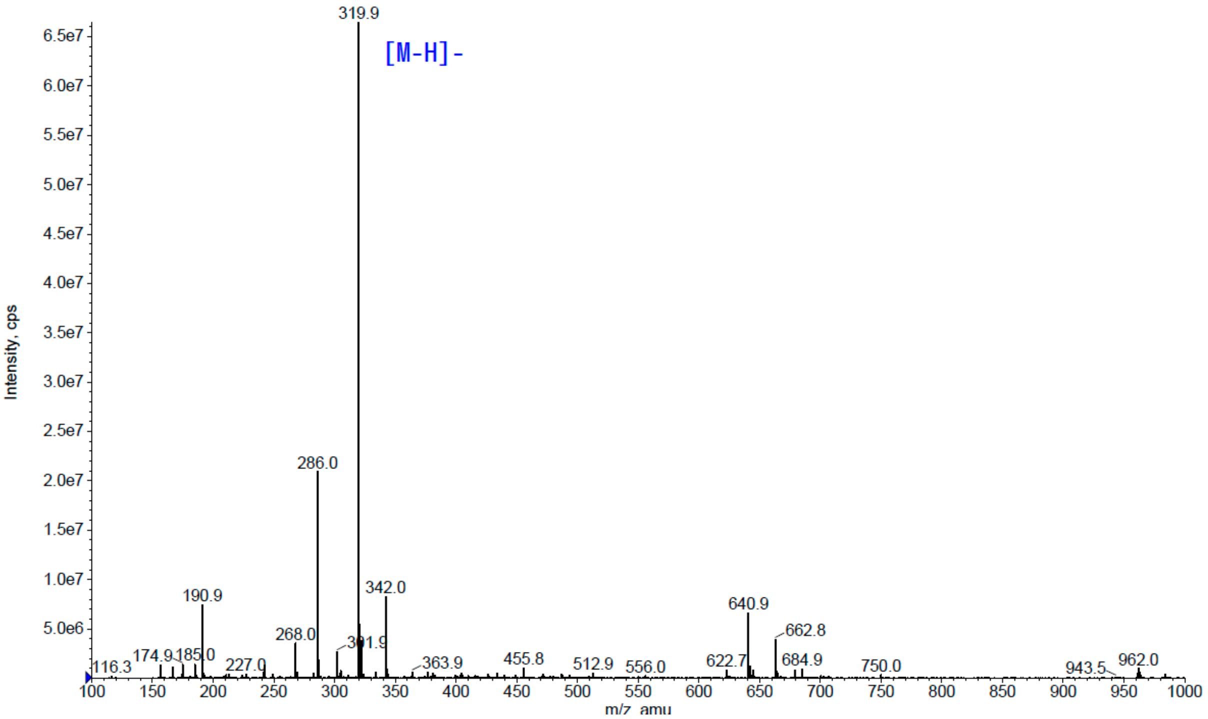

Figure S22: ESI-MS data of crude peptide **1.70**

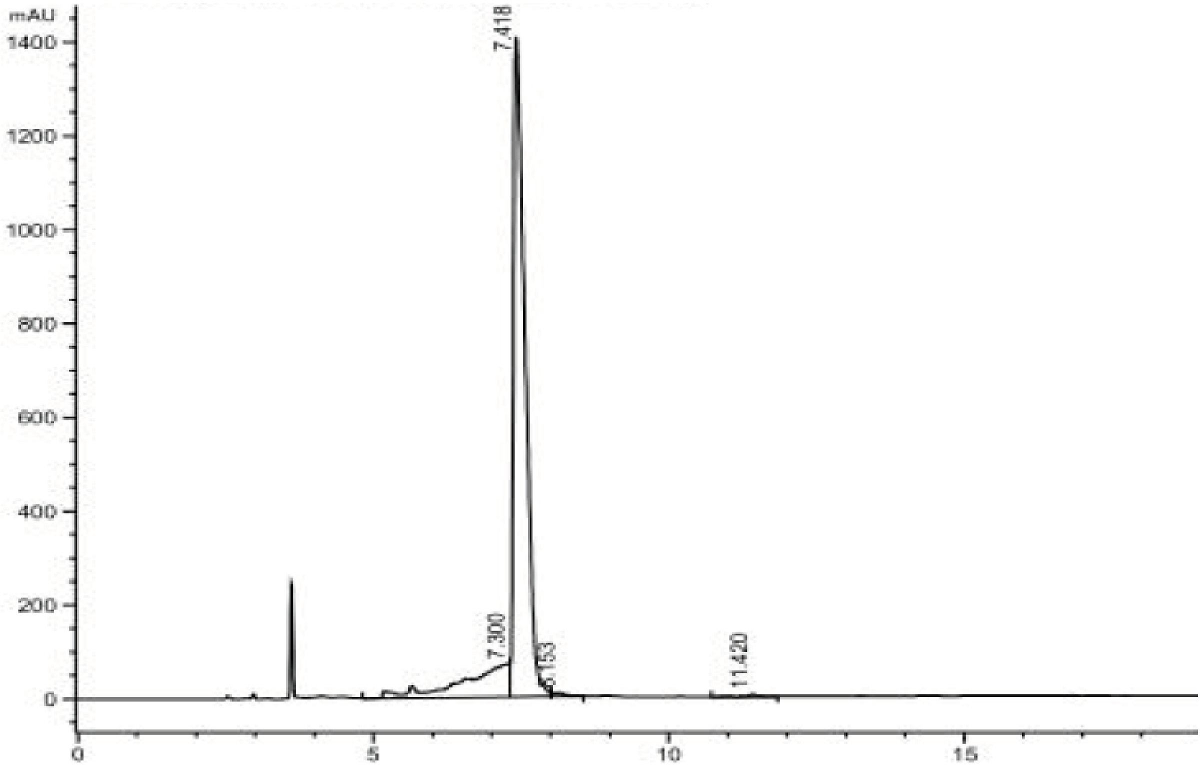

Figure S23: HPLC chromatogram of crude peptide **1.70**

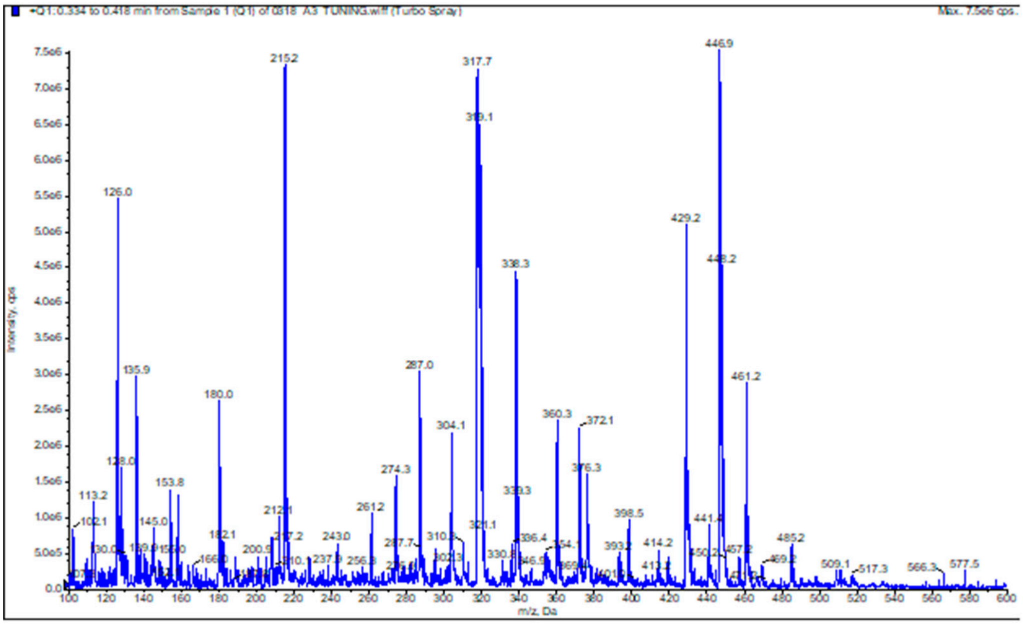

Figure S24: LC-MS Q1 scan of analogue **1.70**

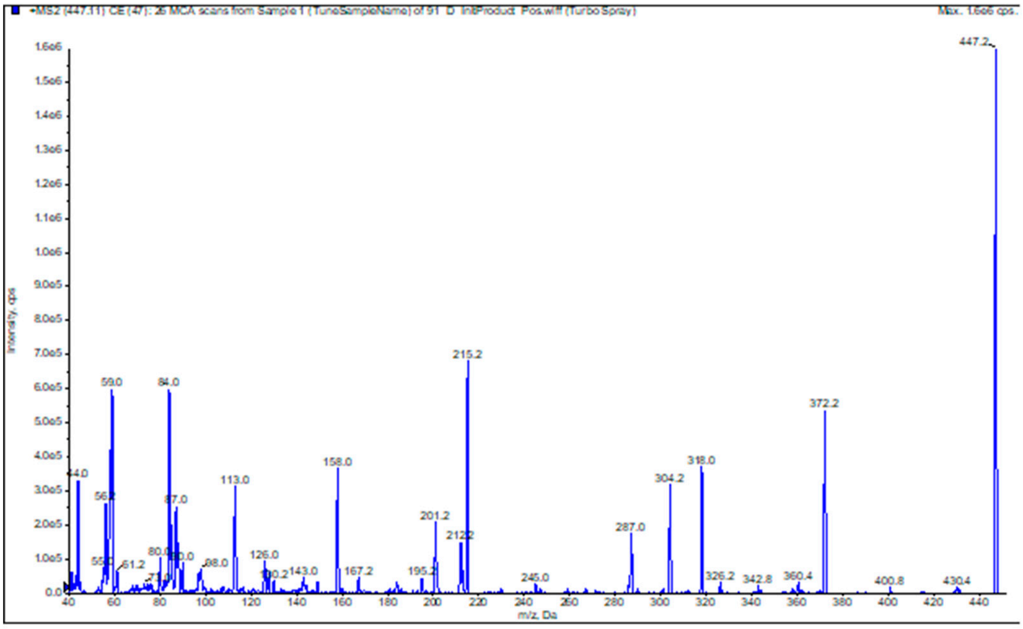

Figure S25: LC-MS Q3 scan of analogue **1.70**

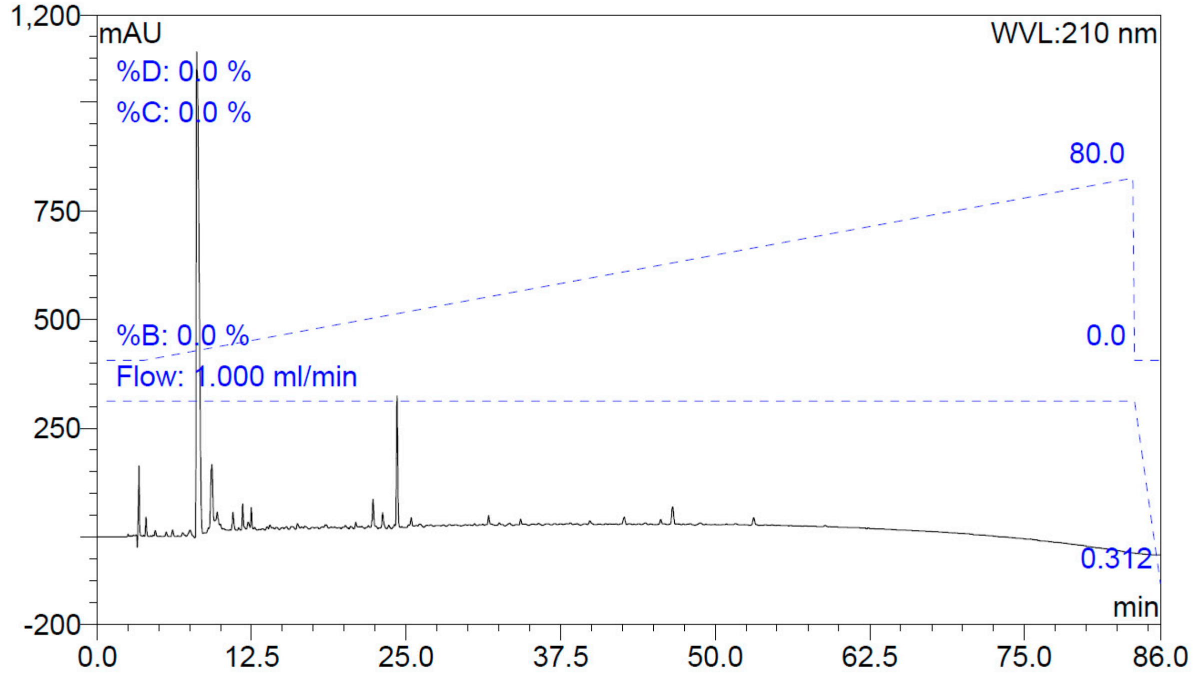

Figure S26: HPLC chromatogram of crude peptide **1.62**
